# Integrated online HILIC-ESI-HRMS and ICP-MS/MS for chemical species profiling in transgenic soybean callus exposed to copper nanoparticles

**DOI:** 10.1007/s00216-026-06596-x

**Published:** 2026-06-13

**Authors:** Raimundo Rafael Gamela, Elisânia Kelly Barbosa Fonseca, Vinnícius Henrique Cerqueira da Silva, Cristiane Renata Schmitt, Marco Aurélio Zezzi Arruda

**Affiliations:** 1 Spectrometry, Sample Preparation and Mechanization Group, Institute of Chemistry, University of Campinas (Unicamp), Campinas, São Paulo 13083-970 Brazil; 2 National Institute of Science and Technology in Bioanalytics – Lauro Kubota (INCTBio-LK), Institute of Chemistry, University of Campinas – Unicamp, P.O. Box 6154, Campinas, São Paulo 13083-970 Brazil

**Keywords:** Nanobiotechnology, Soybean calluses, Metallic nanoparticles, Speciomics analysis

## Abstract

Soybean calluses constitute a robust in vitro model for investigating biochemical and elemental responses to external stressors. In this study, transgenic soybean calluses (*Glycine max*) were exposed to increasing concentrations of copper nanoparticles (CuNPs; 10, 50, and 100 µg L⁻^1^), and their chemical responses were investigated using an integrated online HILIC-ESI-HRMS-ICP-MS/MS platform. This hyphenated analytical approach enabled the simultaneous chromatographic separation, molecular characterization, and elemental detection of co-eluting chemical species, providing complementary metabolomic and speciation information within a single analytical workflow. The online coupling revealed marked concentration-dependent effects, with calluses exposed to 10 and 50 µg L^−1^ CuNPs exhibiting reduced chemical diversity compared with those exposed to 100 µg L^−1^. Multivariate and pathway-level analyses indicated that CuNP exposure progressively reshaped the metabolic landscape, with the most pronounced alterations observed at the highest concentration. These changes were primarily associated with cellular defense responses, redox imbalance, and membrane remodeling, as evidenced by the increased accumulation of oxylipins and lysophospholipids, including 12(13)-Ep-9-KODE, (9Z,12Z)-7,8,16-trihydroxyoctadeca-9,12-dienoic acid, and LysoPC(16:0/0:0). Importantly, the integrated speciomics strategy afforded by the online HILIC-ESI-HRMS-ICP-MS/MS platform enabled the putative annotation of metal- and heteroatom-containing species, suggesting homeostatic adjustments involving essential elements such as Mn, Mg, Zn, P, and S associated with organic ligands. These findings highlight the central role of elemental redistribution and coordination chemistry in shaping the biochemical response of soybean calluses to nanoparticle-induced stress and demonstrate the analytical potential of integrated molecular and elemental detection for investigating complex biological systems.

**Graphical abstract:**

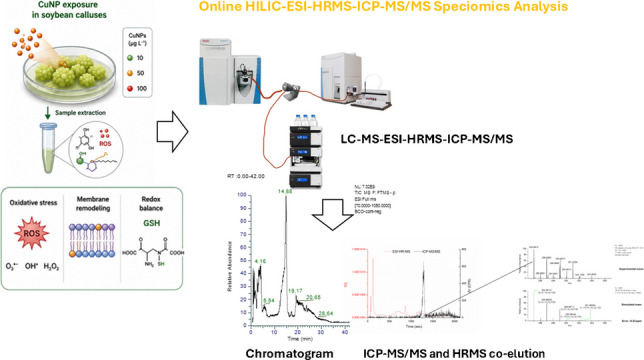

**Supplementary Information:**

The online version contains supplementary material available at 10.1007/s00216-026-06596-x.

## Introduction

Modern agriculture increasingly relies on technological innovations to balance productivity with environmental stress mitigation, particularly in high-input cropping systems such as soybean cultivation [1]. In this context, plant biotechnology and in vitro culture systems have emerged as powerful experimental platforms for investigating cellular responses to environmental stimuli and advancing precision agriculture strategies [2].

Soybean (*Glycine max*) is one of the most economically important crops worldwide and is characterized by a highly dynamic metabolism that plays a central role in stress tolerance, redox regulation, and productivity [3, 4]. Its well-established transformation protocols and adaptability to in vitro cultivation make soybean an excellent model for biotechnological studies, particularly those involving callus tissues [5–7]. In transgenic soybean calluses, the genetically modified metabolic background introduces an additional layer of complexity, providing a valuable system for investigating how altered plant metabolism responds to external perturbations at the molecular level [8].

In recent years, agricultural research has shown growing interest in nanotechnology, particularly in the application of metallic nanoparticles as antimicrobial agents, growth regulators, and modulators of plant stress responses. Among these materials, copper nanoparticles (CuNPs) have attracted particular attention because of the essential role of copper in plant metabolism, redox homeostasis, and enzymatic activity [7–10]. Despite their promising applications, the behavior, transformation, and biological effects of nanoparticles in plant systems remain insufficiently understood, especially with respect to their chemical speciation and interactions with endogenous biomolecules.

Callus cultures represent a robust in vitro model for investigating stress-induced metabolic reprogramming, including alterations in oxidative balance, nutrient regulation, hormone signaling, and secondary metabolism. In systems exposed to metallic nanoparticles, cellular responses are intrinsically linked to the chemical species present, which ultimately determine bioavailability, reactivity, and biological effects [5, 9, 10]. However, studies explicitly combining plant callus cultures with metallic nanoparticle exposure while simultaneously addressing chemical speciation and molecular-level transformations remain scarce. This knowledge gap highlights the need for integrative analytical strategies capable of resolving both molecular and elemental information within complex biological matrices.

Most current studies still rely on either molecular or elemental analyses in isolation, limiting the interpretation of nanoparticle-induced biochemical responses. Classical approaches such as high-performance liquid chromatography coupled to inductively coupled plasma mass spectrometry (HPLC-ICP-MS) provide excellent sensitivity and elemental selectivity and have been widely applied in targeted speciation studies when the analytes of interest are known and appropriate standards are available. However, these approaches provide limited molecular information and are inherently restricted when unknown species or highly complex biological matrices are involved, since conventional ICP-MS does not provide structural or compositional information [11, 12].

To overcome these limitations, liquid chromatography has increasingly been coupled with electrospray ionization high-resolution mass spectrometry (LC-ESI-HRMS) and ICP-MS/MS using both offline and online multimodal strategies. Although offline approaches have been successfully applied in speciation studies, uncertainties regarding the stability of metal- and metalloid-containing complexes during fractionation, storage, and reanalysis remain a significant limitation [13]. In contrast, online hyphenation enables simultaneous chromatographic separation with parallel molecular and elemental detection, a prerequisite for comprehensive speciomics investigations integrating chemical speciation with metabolomics. This approach improves confidence in metabolite annotation through retention time alignment and enhances coverage of metal- and heteroatom-associated metabolites compared with conventional offline workflows. Here, the term speciomics refers to the integrated study of molecular speciation and elemental distribution within biological systems. This analytical framework opens new opportunities for identifying intact molecular forms of elements, including metal-organic complexes, metallobiomolecules, and heteroatom-containing species [11, 14–16].

Within this context, the online coupling of HILIC-ESI-HRMS and ICP-MS/MS [14, 17] provides a powerful multidimensional platform for investigating complex biological systems. High-resolution mass spectrometry enables broad and sensitive coverage of polar and semi-polar metabolites, while ICP-MS/MS allows selective and qualitative detection of metals and non-metals potentially associated with these biomolecules. Together, these complementary techniques support a more robust interpretation of chemical speciation and elemental redistribution, allowing discrimination among ionic, complexed, and biomolecule-associated forms within plant tissues [18].

By leveraging this integrated analytical strategy, stress-induced metabolic reprogramming can be directly correlated with changes in chemical species distribution, providing new insights into how different concentrations of metallic nanoparticles influence cellular homeostasis and coordination chemistry.

Therefore, the present study aimed to investigate the effects of CuNPs on transgenic soybean calluses using an integrated HILIC-ESI-HRMS-ICP-MS/MS speciomics approach. By combining comprehensive molecular profiling with simultaneous elemental detection and speciation analysis, this work provides new insights into CuNP-plant interactions and contributes to the rational evaluation of nanoparticle-based strategies for precision agriculture.

## Materials and methods

### Metallic copper nanoparticles

The metallic CuNPs used in the present study were previously developed and characterized by Schmitt et al. [19]. Their biosynthesis was performed using residual soybean callus tissues as the biological reducing matrix. Physicochemical characterization was carried out using ultraviolet–visible spectroscopy (UV–Vis), dynamic light scattering (DLS), and transmission electron microscopy (TEM), revealing an average particle size of approximately 10 nm, uniform morphology, and high colloidal stability (zeta potential: + 60 mV). Complementary Raman and Fourier transform infrared (FTIR) spectroscopy analyses further confirmed the metallic copper structure (Cu⁰).

### Soybean callus cultivation

Transgenic Roundup Ready soybean seeds (carrying the cp4-EPSPS transgene) were surface-disinfected and subsequently transferred to Murashige and Skoog (MS) culture medium (pH 5.8) containing basal salts supplemented with thiamine, myo-inositol, sucrose, and agar. After 10 days of cultivation, leaf and cotyledonary nodes were excised and used as explants for callus induction. The explants were then transferred to MS medium supplemented with vitamins and the plant growth regulators benzylaminopurine (BAP) and thidiazuron (TDZ) [5].

To evaluate the effects of copper nanoparticles on callus development, three CuNP concentrations (10, 50, and 100 µg L^−1^) were tested. The calluses were maintained in a growth chamber under a 12-h photoperiod at 27 °C for 30 days, after which they were harvested for further analysis.

### Sample preparation

The samples were ground to a fine, homogeneous powder under liquid nitrogen. A subsample of approximately 200 mg was weighed and subjected to extraction with 1.5 mL of acetonitrile/water (50:50, v/v). The mixture was vortexed for 1 min and subsequently sonicated using a Qsonica ultrasonic processor (Newtown, USA) for 90 s using a 10-s on/off pulse cycle. The extracts were then centrifuged at 8500 rpm for 10 min, and the resulting supernatants were filtered through a 0.22-µm membrane filter and transferred to amber vials for analysis.

To minimize experimental variability, all calluses were cultivated under identical environmental conditions, and samples were processed using a standardized extraction and analytical workflow. Despite these precautions, variations in extraction efficiency and ionization response, particularly for metal-associated chemical species, may influence signal intensity. Therefore, the present study emphasizes the reproducibility of metabolic trends and pathway-level modulation rather than absolute quantitative reproducibility.

### Species separation

Chromatographic separation was performed using a Thermo Scientific Ultimate 3000 system (Waltham, MA, USA) equipped with a cooled autosampler. Separation was achieved using an Accucore Amide-HILIC column (250 × 2.1 mm, 2.6 µm; Thermo Scientific) operated under gradient elution at a flow rate of 0.750 mL min^−1^. The mobile phases consisted of (A) acetonitrile and (B) 5 mM ammonium formate containing 0.1% (v/v) formic acid (pH 5.5).

The gradient program was as follows: 0–2.5 min, 10% B; 2.5–22.5 min, linear increase to 50% B; 22.5–25.0 min, 50% B; 25.0–26.0 min, increase to 65% B; 26.0–27.5 min, 65% B; and 27.5–30.0 min, return to 10% B for column re-equilibration.

A 15-µL aliquot of each extract was injected onto the HILIC column, which was maintained at 35 °C. After chromatographic separation, the column effluent was directed through a Series 600 flow splitter (ASI Company, USA), splitting the flow such that 30% was directed to the ICP-MS/MS and 70% to the ESI-HRMS.

#### Identification of chemical species using HILIC-ESI-HRMS-ICP-MS/MS

An iCAP TQ ICP-MS/MS (Thermo Scientific, Bremen, Germany) was used for the determination of the monitored elements, including iron (Fe), copper (Cu), zinc (Zn), molybdenum (Mo), nickel (Ni), magnesium (Mg), manganese (Mn), calcium (Ca), sulfur (S), and phosphorus (P). Oxygen (O_2_; 30%, 0.3 mL min^−1^) and hydrogen (H_2_; 1.1 mL min^−1^) were used as reaction gases in MS/MS mode. The ICP-MS/MS was operated in organic mode using platinum cones (Pt cones), and the sample introduction system consisted of a micro-PFA nebulizer with supplemental oxygen/argon (15:85, v/v) added to the nebulizer gas. The influence of the acetonitrile gradient on the ICP-MS/MS signal was monitored as previously described by Kato et al. [14].

The instrument was operated using 99.999% argon (White Martins-Praxair, São Paulo, Brazil), with an RF power of 1500 W, plasma gas flow of 14 L min^−1^, and nebulizer gas flow of 0.99 mL min^−1^. Because an organic interface was employed, the spray chamber temperature was maintained at −2 °C.

An Orbitrap Q Exactive HRMS (Thermo Scientific, Bremen, Germany), equipped with a heated electrospray ionization (HESI) source, was used for molecular identification of eluting compounds in parallel with the ICP-MS/MS system. The instrument was operated in both positive and negative ionization modes with a spray voltage of 3.5 kV and a resolving power of 70,000 (at m/z 200), over a scan range of m/z 70–1050.

Data acquisition consisted of full-scan MS followed by MS/MS analysis in data-dependent acquisition (DDA) mode, targeting the five most intense precursor ions detected in the HRMS signal. Stepped normalized collision energies of 20, 30, and 50 eV were applied.

Arsenobetaine (m/z 179.004) was used as an internal standard because it allowed simultaneous detection of arsenic by ICP-MS/MS (m/z 91, monitored as ^75^As^16^O in mass-shift mode) and its corresponding molecular ion (C_5_H_12_AsO_2_, m/z 179.004) by HRMS at the same retention time. Each analytical sequence began with blank injections (mobile phase and extraction solvent only), followed by sample analysis.

Compound identification and confirmation of fragmentation patterns were performed using Compound Discoverer (Thermo Scientific), with ChemSpider, mzCloud, and MassBank as reference databases. The following adducts were considered during data processing: [M+H]⁺, [M+Na]⁺, [M+K]⁺, [M+NH₄]⁺, [M+CH₃OH+H]⁺, [M+ACN+H]⁺, and [M+H−H₂O]⁺ in positive mode; and [M−H]⁻, [M+Cl]⁻, [M−H₂O−H]⁻, [M+Na−2H]⁻, [M+K−2H]⁻, and [M+FA−H]⁻ in negative mode.

Chemometric analysis, including principal component analysis (PCA), was performed using the MetaboAnalyst 6.0 platform.

## Results and discussions

### Assessment of transgenic soybean callus after cultivation

**Fig. 1:**
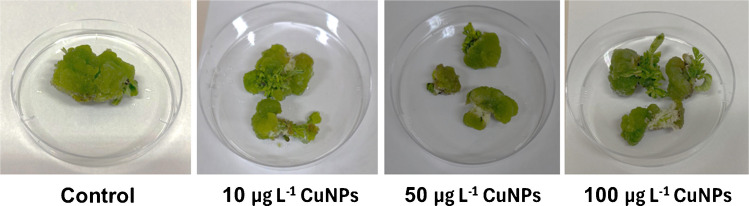
Transgenic soybean calluses cultured after 30 days under control conditions and exposed to CuNPs (10, 50 and 100 µg L^−1^)

Figure 1 shows representative transgenic soybean calluses derived from Roundup Ready soybean and cultivated for 30 days under the different experimental conditions. As the objective of this study was to evaluate the effects of CuNPs on callus tissues during this period, only macroscopic physiological parameters, including callus development, oxidation status, and coloration, were assessed.

Regarding callus development, normal growth was observed under all experimental conditions, including exposure to 10, 50, and 100 µg L^−1^ of CuNPs, as well as in the control treatment (absence of CuNPs). All calluses exhibited a healthy green color, indicating tissue viability and the absence of visible oxidative stress symptoms, consistent with previous observations reported by da Silva and Arruda [6]. No visible growth inhibition was observed in any of the treatments.

Importantly, the absence of macroscopic physiological alterations suggests that CuNP-induced responses occur primarily at the molecular and metabolic levels. This finding reinforces the relevance of the integrated HILIC-ESI-HRMS-ICP-MS/MS approach employed in this study, which enables the detection of early and subtle biochemical reprogramming events that are not detectable by visual inspection.

### Chemical species profile and comparative analysis of transgenic soybean calluses

#### Multivariate analysis of chemical species

To obtain an overview of the chemical species variation induced by CuNP exposure, an unsupervised principal component analysis (PCA) was applied to the dataset generated by the integrated online HILIC-ESI-HRMS-ICP-MS/MS platform. PCA was used as an exploratory multivariate approach to evaluate global similarities and differences among samples and to identify the main sources of variability within the chemical species dataset [20].

The PCA model showed that the first two principal components captured a substantial proportion of the total variance, with PC1 explaining 40.2% and PC2 accounting for 37.2%, together representing 77.4% of the overall dataset variability. This high cumulative variance indicates that the two-dimensional PCA model provides a robust overview of the main metabolic alterations induced by CuNP exposure in soybean calluses.

**Fig. 2:**
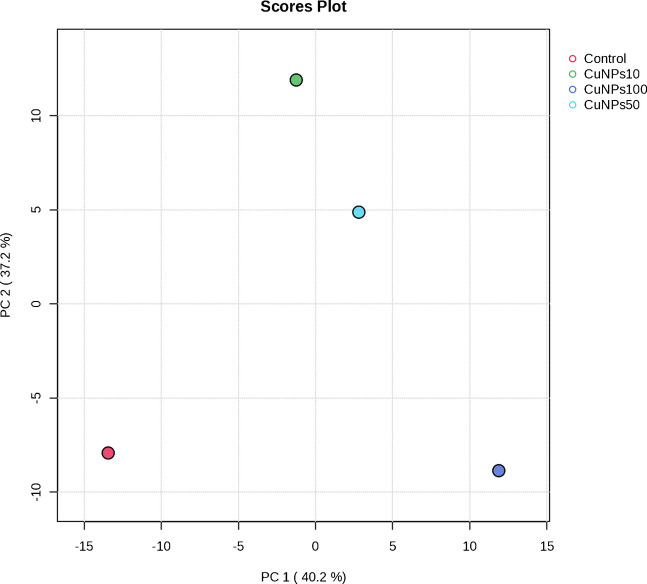
PCA score plot of chemical species profiles obtained from transgenic soybean calluses exposed to different concentrations (control, 10, 50, and 100 µg L^−1^) of CuNPs

The PCA score plot (Fig. 2) revealed a clear separation among the experimental groups, indicating a concentration-dependent metabolic reorganization. Control calluses were distinctly positioned in the negative region of both PC1 and PC2, reflecting the basal metabolic profile characteristic of unstressed tissues. In contrast, all CuNP-treated samples shifted toward positive PC1 values, indicating that this component primarily captured the global metabolic response associated with nanoparticle exposure.

Among the treated groups, calluses exposed to 10 µg L^−1^ CuNPs were mainly distributed along positive PC2 values while remaining close to the PC1 origin, suggesting early adaptive metabolic adjustments rather than extensive metabolic reprogramming. The 50 µg L^−1^ treatment occupied an intermediate position, displaced toward positive PC1 and moderately positive PC2 values, consistent with a progressive intensification of stress-related metabolic responses. Notably, calluses exposed to 100 µg L^−1^ CuNPs formed a clearly distinct cluster characterized by strongly positive PC1 and negative PC2 scores, indicating a pronounced and qualitatively different metabolic state at the highest nanoparticle concentration.

**Fig. 3:**
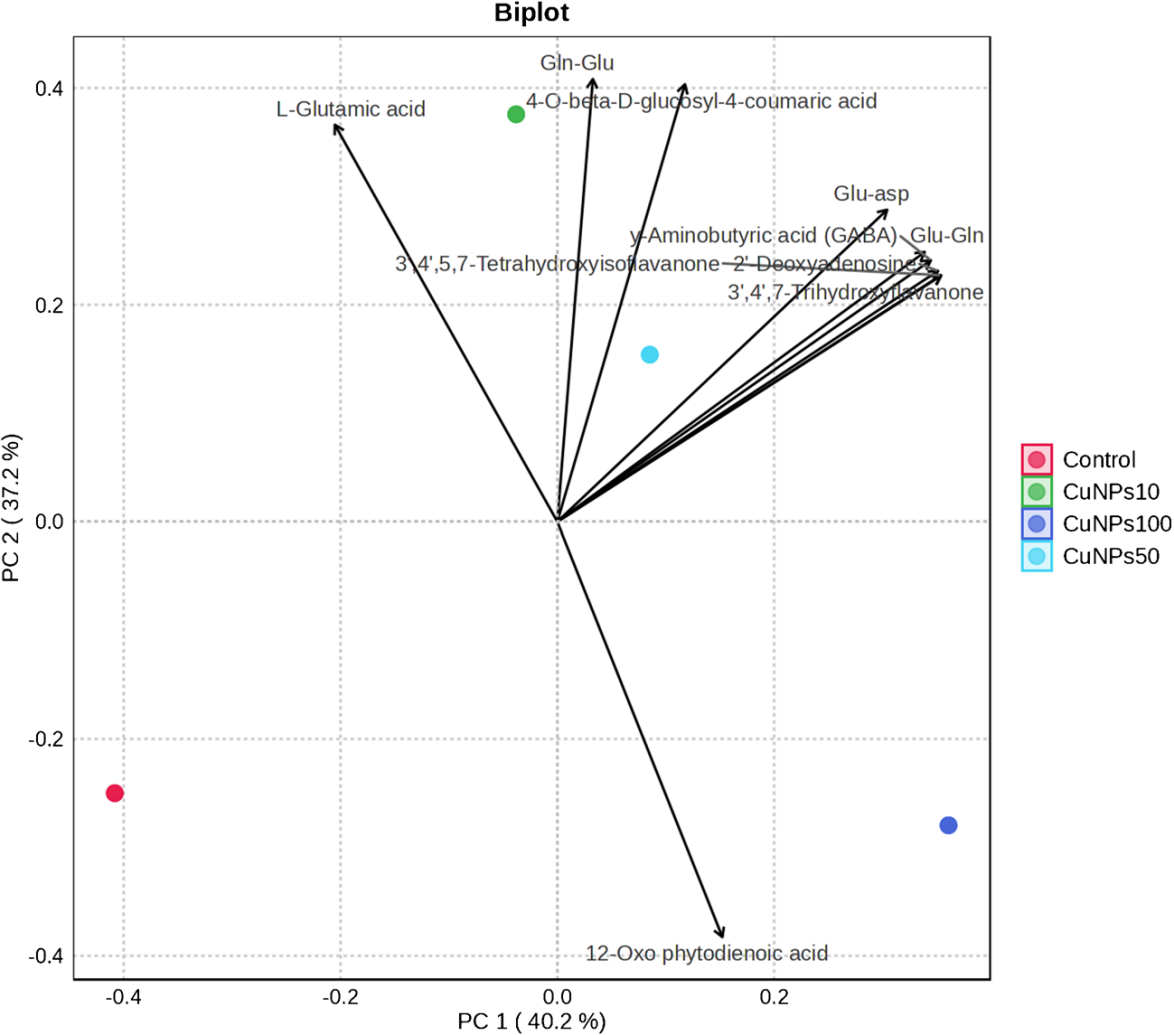
PCA loading plot highlights chemical species contributing to sample discrimination in soybean calluses exposed to CuNPs

To identify the chemical species contributing to these separations, the PCA biplot was further examined (Fig. 3). Positive PC1 loadings were predominantly associated with stress- and defense-related metabolites, including γ-aminobutyric acid (GABA), dipeptides such as Glu–Gln and Glu–Asp, deoxynucleosides, flavanone derivatives, and phenylpropanoid-related compounds such as 4-O-β-D-glucosyl-4-coumaric acid. Their close alignment with CuNP-treated samples indicates a major contribution to the observed nanoparticle-induced metabolic shift.

In contrast, negative PC1 loadings were mainly associated with metabolites involved in primary metabolism and nitrogen assimilation, particularly L-glutamic acid, which was closely aligned with the control group, reinforcing its role as a marker of the basal metabolic state.

PC2 provided additional discrimination among CuNP concentrations. Positive PC2 loadings were associated with amino acid–related metabolites and compounds involved in adaptive and buffering responses, contributing to the positioning of the 10 and 50 µg L^−1^ treatments. In contrast, strong negative PC2 loadings were dominated by 12-oxo-phytodienoic acid, an oxylipin precursor involved in jasmonate signaling and oxidative stress responses. Its close association with the 100 µg L⁻^1^ treatment suggests enhanced activation of lipid-derived signaling pathways and a more severe oxidative stress condition at the highest CuNP concentration.

Despite the clear separation observed in both the PCA scores and biplot, it is important to recognize that PCA primarily captures global variance patterns driven by the most abundant and co-varying metabolites. Consequently, some chemical species detected exclusively in calluses exposed to 100 µg L^−1^ CuNPs did not contribute substantially to group separation, as PCA inherently emphasizes variables explaining the largest proportion of total variance, while condition-specific or low-abundance metabolites may exert limited influence on the principal components.

Overall, the combined interpretation of PCA scores and loadings indicates a transition from basal metabolism in control calluses to adaptive metabolic reorganization at low and intermediate CuNP concentrations, culminating in pronounced stress-driven metabolic reprogramming at 100 µg L^−1^. It should be emphasized that PCA was applied exclusively as an unsupervised exploratory tool to visualize global metabolic trends and major sources of variability. No supervised classification or predictive modeling was performed, and the PCA results are presented solely to support the pathway-level interpretations discussed in the following sections.

#### Trends in chemical species diversity and chemical classes composition

When comparing the chemical species profiles across the four experimental conditions, a total of 118 species were identified in transgenic soybean calluses cultured in the absence of CuNPs (control). In contrast, exposure to CuNPs resulted in a reduction in the number of detected species at lower concentrations, with 96 and 94 species identified at 10 and 50 µg L^−1^, respectively.

Overall, a non-linear, concentration-dependent pattern was observed. Calluses exposed to 10 µg L^−1^ CuNPs exhibited an approximately 18% reduction in detectable chemical species relative to the control, indicating decreased metabolic complexity. At 50 µg L^−1^, the number of detected species remained similar to that observed at the lower concentration, suggesting maintenance of a restricted metabolic profile rather than metabolic expansion. In contrast, calluses exposed to 100 µg L^−1^ CuNPs showed a clear increase in chemical diversity, reaching 122 detected species—exceeding the number observed in the control group. This increase indicates a marked metabolic shift relative to the lower CuNP concentrations.

**Fig. 4:**
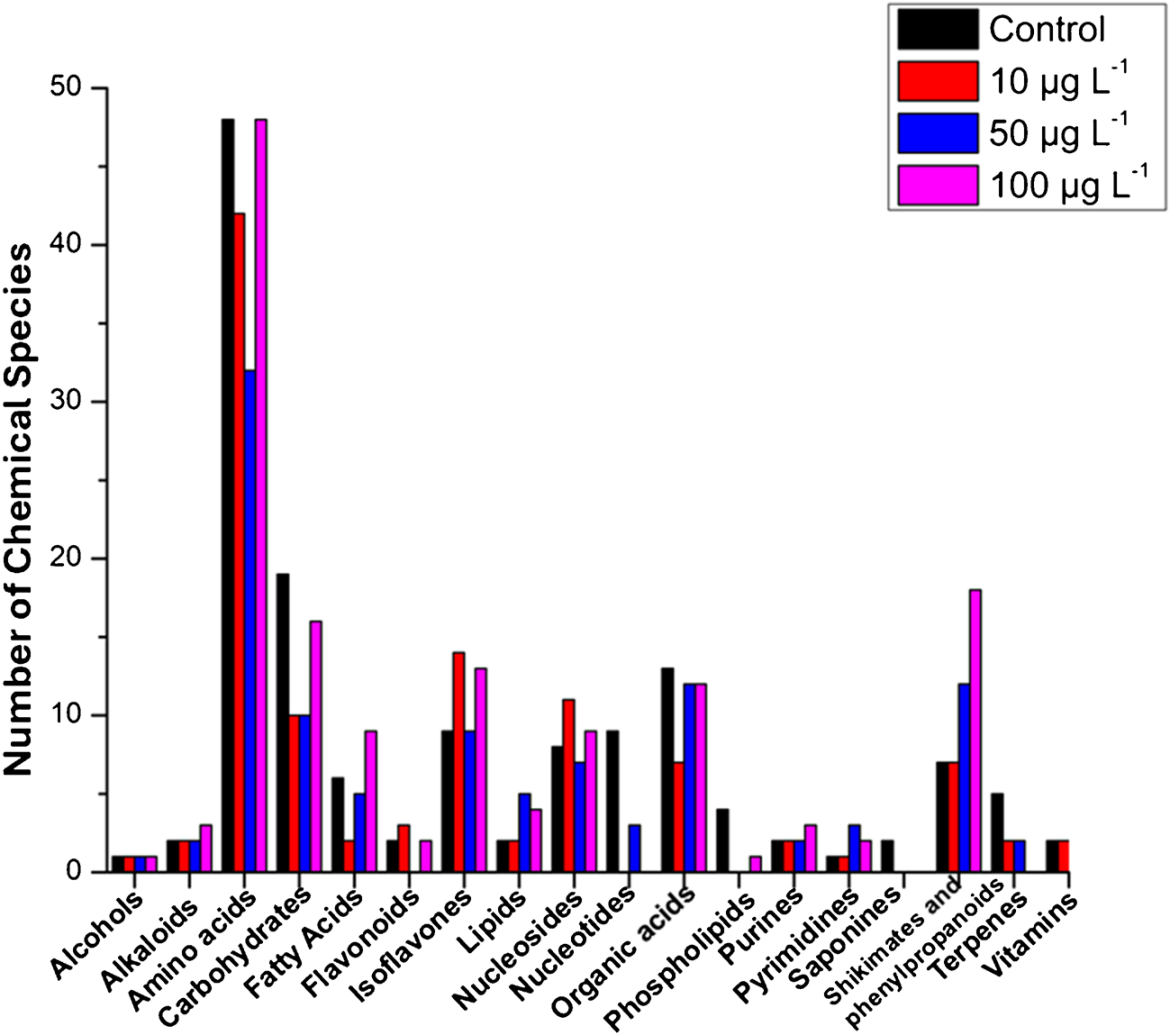
Chemical species classes identified in transgenic soybean calluses exposed to different concentrations of CuNPs

With respect to chemical classes (Fig. 4), amino acids represented the most abundant class across all treatments, followed by carbohydrates and shikimate-related compounds. These three classes showed reduced representation at 10 and 50 µg L^−1^ and increased abundance at 100 µg L^−1^. Isoflavones, organic acids, nucleosides, and nucleotides were detected at intermediate levels, whereas lipids and saponins were less abundant and exhibited only modest variation among treatments.

Importantly, most of the identified metabolite classes have been previously reported in soybean tissues and are associated with central metabolic pathways, including carbon, nitrogen, carbohydrate, amino acid, and organic acid metabolism, reinforcing the biological relevance of the detected chemical profiles [21–27].

### Chemical species response and metabolic pathway induced by CuNPs in transgenic soybean calluses cultivation

Table 1 summarizes the chemical species identified in transgenic soybean calluses exposed to 100 µg L^−1^ CuNPs, based on annotation using the ChemSpider, mzCloud, and MassBank databases. Additional information on the chemical species identified under control conditions (absence of CuNPs) and after exposure to 10 and 50 µg L^−1^ CuNPs is provided in Supplementary Tables S1, S2, and S3, respectively.

**Table 1:** Chemical species identified in transgenic soybean calluses cultured at 100 µg L^−1^ of CuNPs using online integrated HILIC-ESI-HRMS-ICP-MS/MS

Chemical species	Formulae	Annot. DeltaMass (ppm)	Calc. MW	m/z	Reference ion	RT (min)
Indole	C8 H7 N	−0.08	117.0578	118.0651	[M+H]+1	2.45
Trans-3-indoleacrylic acid	C11 H9 N O2	−0.5	187.0632	188.0705	[M+H]+1	4.28
Trigonelline	C7 H7 N O2	−0.61	137.0476	138.0549	[M+H]+1	9.88
y-Aminobutyric acid (GABA)	C4 H9 N O2	−0.5	103.0633	86.06001	[M+H−H2O]+1	19.81
y-Glu-Ala	C8 H14 N2 O5	−1.41	218.09	217.0827	[M−H]−1	7.23
2-Ureidoglycine	C3 H7 N3 O3	−0.48	133.0487	134.056	[M+H]+1	15.37
3-Hydroxynorvaline	C5 H11 N O3	−0.64	133.0738	134.0811	[M+H]+1	23.19
4-Guanidinobutyric acid	C5 H11 N3 O2	−0.29	145.0851	146.0924	[M+H]+1	18.29
Allantoic acid	C4 H8 N4 O4	−1.17	176.0544	215.0175	[M+K]+1	15.38
Allantoin	C4 H6 N4 O3	−0.2	158.044	157.0367	[M−H]−1	2.07
Aminoadipic acid	C6 H11 N O4	−0.24	161.0688	160.0615	[M−H]−1	2.73
Asn-Ile-Leu	C16 H30 N4 O5	−11.62	358.2175	339.1996	[M−H−H2O]−1	0.66
Asn-Thr-Tyr	C17 H24 N4 O7	−4.44	396.1627	435.1259	[M+K]+1	2.67
Asp-Glu	C9 H14 N2 O7	−1.53	262.0797	263.087	[M+H]+1	23.35
Cys-pro	C8 H14 N2 O3 S	−1.41	218.0722	219.0795	[M+H]+1	1.56
L-Proline	C5 H9 N O2	−0.16	115.0633	116.0706	[M+H]+1	10.54
D-a-Aminobutyric acid (PABA)	C4 H9 N O2	0.38	103.0634	102.0561	[M−H]−1	21.47
L-Glutamine	C5 H10 N2 O3	−0.22	146.0691	145.0618	[M−H]−1	7.43
DL-Pyroglutamic acid	C5 H7 N O3	−0.06	129.0426	128.0353	[M−H]−1	2.13
Gamma-Glutamyl-2-aminobutyric acid	C9 H16 N2 O5	−1.68	232.1055	233.1128	[M+H]+1	23.53
Gamma-Glutamylisoleucine	C11 H20 N2 O5	−1.98	260.1367	261.144	[M+H]+1	23.15
Gamma-Glutamyltyrosine	C14 H18 N2 O6	0.26	310.1166	291.0987	[M−H−H2O]−1	2.54
Gln-Gln	C10 H18 N4 O5	−1.17	274.1274	275.1347	[M+H]+1	24.17
Gln-Glu	C10 H17 N3 O6	0.08	275.1118	274.1045	[M−H]−1	24.52
Glu-Ala	C8 H14 N2 O5	−1.81	218.0899	219.0972	[M+H]+1	23.34
Glu-asp	C9 H14 N2 O7	−0.33	262.08	261.0728	[M−H]−1	23.66
Glu-Gln	C10 H17 N3 O6	−1.51	275.1113	258.108	[M+H−H2O]+1	23.60
Glycylproline	C7 H12 N2 O3	−0.09	172.0848	171.0775	[M−H]−1	19.24
Glycyl-prolyl-glutamic acid	C12 H19 N3 O6	−1.28	301.127	302.1343	[M+H]+1	7.03
Isoleucylproline	C11 H20 N2 O3	−1.36	228.1471	229.1544	[M+H]+1	16.55
L-Asparagine	C4 H8 N2 O3	−1.1	132.0534	133.0606	[M+H]+1	23.33
L-Aspartic acid	C4 H7 N O4	−0.18	133.0375	132.0302	[M−H]−1	22.94
Leu-Val	C11 H22 N2 O3	−1.25	230.1628	231.17	[M+H]+1	5.27
L-Glutamic acid	C5 H9 N O4	1.26	147.0533	146.0461	[M−H]−1	13.44
L-Pyroglutamic acid	C5 H7 N O3	−0.18	129.0426	128.0353	[M−H]−1	1.15
L-Threonine	C4 H9 N O3	−0.31	119.0582	120.0655	[M+H]+1	23.35
N-a-Acetyl-L-arginine	C8 H16 N4 O3	−1.48	216.1219	217.1292	[M+H]+1	19.39
N-Acetylglutamic acid	C7 H11 N O5	−0.94	189.0635	172.0603	[M+H-H2O]+1	7.48
N-Acetyl-L-asparagine	C6 H10 N2 O4	−0.33	174.064	155.0462	[M−H−H2O]−1	8.39
N-Acetyl-L-aspartic acid	C6 H9 N O5	−0.84	175.0479	174.0407	[M−H]−1	9.72
N-Acetyl-L-histidine	C8 H11 N3 O3	−0.59	197.0799	196.0727	[M−H]−1	15.81
O-Acetylserine	C5 H9 N O4	−0.19	147.0531	146.0459	[M−H]−1	5.10
Pro-Lys-Ser	C14 H26 N4 O5	−11.72	330.1865	311.1686	[M−H−H2O]−1	0.66
S-Methyl-L-cysteine-S-oxide	C4 H9 N O3 S	−0.76	151.0302	152.0375	[M+H]+1	5.39
D-Fructose	C6 H12 O6	−0.33	180.0633	179.0561		3.99
D-Raffinose pentahydrate	C18 H32 O16	−0.62	504.1687	543.1318	[M+K]+1	21.29
D-Xylulose	C5 H10 O5	−1.11	150.0527	149.0454	[M−H]−1	3.86
Galactitol	C6 H14 O6	−0.86	182.0789	181.0717	[M−H]−1	5.46
I-Inositol	C6 H12 O6	−0.18	180.0634	179.0561	[M−H]−1	16.90
L-Arabinitol	C5 H12 O5	−0.02	152.0685	133.0506	[M−H−H2O]−1	2.17
Maltotriose	C18 H32 O16	−0.76	504.1687	503.1614	[M−H]−1	25.37
Phosphohexose	C6 H11 O8 P	−0.78	242.019	241.0117	[M−H]−1	19.77
ß-D-Glucopyranuronic acid	C6 H10 O7	0.05	194.0427	193.0354	[M−H]−1	17.72
Sucrose	C12 H22 O11	−0.66	342.116	377.0854	[M+Cl]−1	20.62
(9Z.12Z)−7.8.16-trihydroxyoctadeca-9.12-dienoic acid	C18 H32 O5	−0.22	328.2249	309.2071	[M−H−H2O]−1	0.84
13(S)-HOTrE	C18 H30 O3	0.08	294.2195	293.2123	[M−H]−1	0.79
13-OxoODE	C18 H30 O3	−1.12	294.2192	295.2264	[M+H]+1	0.86
8-{3-Oxo-2-[(2E)−2-penten-1-yl]−1-cyclopenten-1-yl}octanoic acid	C18 H28 O3	−1.46	292.2034	293.2107	[M+H]+1	0.91
Tetradecanedioic acid	C14 H26 O4	0.41	258.1832	259.1905	[M+H]+1	0.75
(-)-Epigallocatechin 3-(4-methyl-gallate)	C23 H20 O11	−0.69	472.1002	471.093	[M−H]−1	7.16
5.7-Dihydroxy-6-[3.4.5-trihydroxy-6-(hydroxymethyl)oxan-2-yl]chromen-4-one	C15 H16 O9	−1.37	340.079	363.0682	[M+Na]+1	6.05
3′,4′,5,7-Tetrahydroxyisoflavanone	C15 H12 O6	−1.22	288.063	289.0703	[M+H]+1	3.59
3′,4′,7-Trihydroxyflavanone	C15 H12 O5	−1.14	272.0682	273.0754	[M+H]+1	4.40
5-Deoxycajanin	C16 H12 O5	−1.31	284.0681	285.0754	[M+H]+1	0.85
7-Hydroxy-2-(4-hydroxyphenyl)−4-oxo-3.4-dihydro-2H-chromen-5-yl ß-D-glucopyranoside	C21 H22 O10	−0.25	434.1212	433.1139	[M−H]−1	1.26
Coumestrol	C15 H8 O5	−0.42	268.0371	267.0298	[M−H]−1	1.58
Daidzein	C15 H10 O4	−1.5	254.0575	255.0648	[M+H]+1	17.50
Daidzein-4′-glucoside	C21 H20 O9	−1.46	416.1101	417.1174	[M+H]+1	1.04
Genistein	C15 H10 O5	0.29	270.0529	269.0456	[M−H]−1	2.36
Malonyldaidzin	C24 H22 O12	−0.96	502.1106	503.1179	[M+H]+1	17.95
Malonylgenistin	C24 H22 O13	−1.53	518.1053	519.1125	[M+H]+1	1.48
3-O-ß-D-galactosyl-sn-glycerol	C9 H18 O8	−0.9	254.0999	253.0928	[M−H]−1	7.50
LysoPC(16:0/0:0)	C24 H50 N O7 P	−0.95	495.332	496.3393	[M+H]+1	4.36
PC(20:0/P-18:0)	C46 H92 N O7 P	−3.11	801.6586	800.6514	[M−H]−1	14.91
Phytosphingosine	C18 H39 N O3	−1.66	317.2925	318.2997	[M+H]+1	3.53
2′-Deoxyadenosine	C10 H13 N5 O3	−2.01	251.1013	252.1086	[M+H]+1	1.44
Adenosine	C10 H13 N5 O4	−1.01	267.0965	268.1038	[M+H]+1	1.60
Guanosine	C10 H13 N5 O5	−1.68	283.0912	322.0544	[M+K]+1	3.83
Inosine	C10 H12 N4 O5	0.32	268.0809	267.0736	[M−H]−1	2.36
Cyclic ADP-ribose	C15 H21 N5 O13 P2	−0.11	541.0611	540.0538	[M−H]−1	24.52
FAD	C27 H33 N9 O15 P2	0.37	785.1574	784.1502	[M−H]−1	21.50
UDP-GlcNAc	C17 H27 N3 O17 P2	−0.22	607.0814	606.0742	[M−H]−1	23.18
Uridine	C9 H12 N2 O6	−0.14	244.0695	243.0622	[M−H]−1	1.66
2-Hydroxyadipic acid	C6 H10 O5	−1.34	162.0526	325.1125	[2M+H]+1	7.70
Citric acid	C6 H8 O7	0.02	192.027	191.0197	[M−H]−1	23.02
Dehydroascorbic acid	C6 H6 O6	−0.86	174.0163	173.009	[M−H]−1	1.51
Gluconic acid	C6 H12 O7	−0.86	196.0581	195.0509	[M−H]−1	18.88
Pyruvic acid	C3 H4 O3	0.07	88.01605	87.00877	[M−H]−1	3.94
Succinic acid semialdehyde	C4 H6 O3	−0.03	102.0317	101.0244	[M−H]−1	5.44
1-(sn-glycero-3-phospho)−1D-myo-inositol	C9 H19 O11 P	−0.32	334.0664	333.0591	[M−H]−1	23.01
1-Methyladenine	C6 H7 N5	−0.61	149.0701	150.0773	[M+H]+1	7.28
Adenine	C5 H5 N5	−0.67	135.0544	136.0617	[M+H]+1	1.55
Guanine	C5 H5 N5 O	−0.63	151.0493	152.0566	[M+H]+1	2.77
1-Methylcytosine	C5 H7 N3 O	−0.21	125.0589	126.0662	[M+H]+1	6.02
Cytosine	C4 H5 N3 O	−0.01	111.0433	112.0505	[M+H]+1	3.13
1,2,3-Trihydroxybenzene	C6 H6 O3	−0.04	126.0317	127.039	[M+H]+1	2.66
1,2,4-Trihydroxybenzene	C6 H6 O3	−0.27	126.0317	125.0244	[M−H]−1	3.97
2,3-Dihydro-1-benzofuran-2-carboxylic acid	C9 H8 O3	0.11	164.0474	163.0401	[M−H]−1	1.93
2-hydroxy-5-[3,4,5-trihydroxy-6-(hydroxymethyl)oxan-2-yl]oxybenzoic acid	C13 H16 O9	0.06	316.0795	315.0722	[M−H]−1	2.54
3-[2-[3,4,5-Trihydroxy-6-(hydroxymethyl)oxan-2-yl]oxyphenyl]prop-2-enoic acid	C15 H18 O8	−0.15	326.1001	361.0695	[M+Cl]−1	2.83
3-Methoxy-4-[3,4,5-trihydroxy-6-(hydroxymethyl)oxan-2-yl]oxybenzoic acid	C14 H18 O9	−1.34	330.0946	353.0839	[M+Na]+1	9.79
4-(beta-D-Glucopyranosyloxy)phenylacetic acid	C14 H18 O8	−1.66	314.0997	337.0889	[M+Na]+1	6.17
4-Aminobenzoic acid (PABA)	C7 H7 N O2	−0.73	137.0476	138.0549	[M+H]+1	1.11
4-Hydroxycinnamic acid	C9 H8 O3	−0.13	164.0473	163.04	[M−H]−1	2.78
4−Methylcatechol	C7 H8 O2	−0.1	124.0524	142.0862	[M+NH4]+1	2.45
Homogentisic acid	C8 H8 O4	−0.58	168.0422	169.0494	[M+H]+1	3.35
Shikimic acid	C7 H10 O5	0.79	174.053	173.0457	[M−H]−1	8.25
D-Pantothenic acid	C9 H17 N O5	−1.61	219.1103	220.1175	[M+H]+1	2.23
Nicotinamide	C6 H6 N2 O	−0.18	122.048	123.0553	[M+H]+1	18.03
Pyridoxal	C8 H9 N O3	−0.27	167.0582	150.0549	[M+H−H2O]+1	2.90

To better understand the biochemical impact of CuNP exposure, the metabolic responses of soybean calluses were interpreted by grouping altered metabolites into major functional pathways, with particular emphasis on the comparison between control samples and those exposed to 100 µg L^−1^ CuNPs. Overall, comparative analysis across all experimental conditions (Table 2) revealed pronounced metabolic alterations, demonstrating that soybean calluses exhibit distinct concentration-dependent biochemical responses to CuNP exposure.

**Table 2:** Comparative analysis of chemical species in transgenic soybean calluses under different CuNPs concentrations based on HILIC-ESI-HRMS-ICP-MS/MS data

Chemical species	**CuNPs**			
	**Control**	**10 µg L**^**−1**^	**50 µg L**^**−1**^	**100 µg L**^**−1**^
y-Aminobutyric acid (GABA)	✓	✓	✓	✓
Pyruvic acid	✓	✓	✓	✓
Lactic acid	✓	X	x	X
L-Valine	✓	X	x	X
Succinic acid semialdehyde	x	X	x	✓
D-a-Aminobutyric acid (PABA)	x	X	x	✓
D-Glyceric acid	✓	✓	✓	X
Cytosine	✓	✓	✓	✓
Xilose	✓	X	x	X
L-Isoleucine	✓	X	x	✓
N-Acetylglycine	✓	X	x	X
Indole	✓	✓	✓	✓
Betaine	✓	X	x	X
L-Threonine	X	X	x	✓
Nicotinamide	✓	X	x	✓
1.2.4-Trihydroxybenzene	X	X	x	✓
1-Methylcytosine	X	X	✓	✓
1.2.3-Trihydroxybenzene	X	X	x	✓
L-Pyroglutamic acid	✓	✓	x	✓
Dihydrothymine	X	X	✓	X
DL-Pyroglutamic acid	x	✓	✓	✓
L-Glutamic acid	✓	✓	✓	✓
D-Ribose	✓	✓	✓	X
D-Asparagine	✓	X	x	X
L-Aspartic acid	✓	✓	✓	✓
L-Arabinitol	x	X	x	✓
L-Asparagine	x	X	x	✓
2-Ureidoglycine	x	X	x	✓
3-Hydroxynorvaline	x	X	x	✓
Adenine	✓	✓	✓	✓
4-Aminobenzoic acid (PABA)	x	X	✓	✓
Trigonelline	x	✓	✓	✓
4-Methylcatechol	x	X	x	✓
Aminoadipic acid	✓	X	✓	✓
L-Glutamine	✓	✓	✓	✓
O-Acetylserine	✓	✓	✓	✓
4-Guanidinobutyric acid	x	X	x	✓
3-Hydroxycinnamic acid	✓	✓	✓	✓
Mevalonic acid	✓	✓	x	X
D-Xylulose	x	X	x	✓
Pyridoxal	✓	X	✓	✓
1-Methyladenine	x	X	x	✓
5-Hydroxybenzofuran-2(3H)-one	x	X	✓	X
S-Methyl-L-cysteine-S-oxide	x	X	x	✓
Guanine	✓	✓	✓	✓
Protocatehuic acid	✓	X	x	X
L-Histidine	✓	X	x	X
Allantoin	x	X	x	✓
Pimelic acid	x	✓	x	X
Citrulline	x	✓	x	X
4-Hydroxycinnamic acid	x	X	✓	✓
2,3-Dihydro-1-benzofuran-2-carboxylic acid	x	X	x	✓
2-Hydroxycinnamic acid	x	✓	✓	X
Homogentisic acid	x	✓	x	✓
Glycylproline	x	X	x	✓
N-Acetylglutamic acid	✓	✓	✓	✓
Dehydroascorbic acid	x	✓	✓	✓
Cis-Aconitic acid	✓	X	✓	X
Gluconic acid	✓	X	x	✓
Shikimic acid	✓	X	x	✓
N-Acetyl-L-asparagine	✓	✓	✓	✓
N-Acetylornithine	✓	X	x	X
N2-Acetylornithine	x	✓	x	X
N-Acetyl-L-aspartic acid	✓	✓	x	✓
Ascorbic acid	✓	✓	x	X
2-Isopropylmalic acid	✓	X	✓	X
Arginine	✓	X	x	X
Gluconolactone	✓	X	x	✓
D-Fructose	✓	X	x	✓
I-Inositol	✓	X	x	✓
Galactitol	✓	✓	✓	✓
Ala-Asp	✓	X	x	x
N6-Acetyl-L-lysine	✓	X	x	x
Trans-3-Indoleacrylic acid	x	X	x	✓
Citric acid	✓	✓	✓	✓
N-Acetyl-L-histidine	✓	✓	✓	✓
L-Arginine	✓	X	x	x
O-Phosphothreonine	x	X	x	✓
4-Pyridoxic acid	x	✓	x	x
Ala-Ile	x	✓	x	x
L-Tryptophan	x	✓	x	x
2-Methylcitric acid	x	X	✓	x
Glu-Gly	✓	✓	✓	x
N-Acetyl-L-phenylalanine	✓	✓	x	x
Glycerophosphoethanolamine	x	✓	✓	x
Allantoic acid	x	X	x	✓
D-Glucose	x	X	✓	x
y-Glu-Ala	x	✓	✓	✓
N-a-Acetyl-L-arginine	x	X	✓	✓
Glutamyl pyruvate	x	✓	x	x
O-Succinyl-L-homoserine	✓	X	x	x
D-Pantothenic acid	✓	X	x	✓
Cys-pro	x	X	✓	✓
Glu-Ala	✓	X	✓	✓
Thr-Glu-Gln	✓	X	x	x
Traumatic acid	x	X	✓	x
Isoleucylproline	x	X	x	✓
Leu-Val	x	✓	x	✓
Val-Asp	✓	✓	x	x
Leu-Thr	x	✓	x	x
Val-Ile	x	✓	✓	x
Ala-Ala-Ala	✓	X	x	x
gamma-Glutamyl-2-aminobutyric acid	x	✓	✓	✓
Phosphohexose	✓	X	x	✓
Cytidine	✓	X	x	x
Pseudouridine	✓	X	✓	x
Uridine	✓	✓	✓	✓
Pro-gln	✓	X	x	x
Glycerophosphoglycerol	✓	X	✓	x
N-Acetyltryptophan	x	✓	✓	x
Ile-Asp	✓	X	x	x
Val-Glu	✓	✓	x	x
L-?-Glutamyl-L-valine	x	X	✓	x
Val-Met	x	✓	x	x
2′-Deoxyinosine	✓	✓	✓	✓
Deoxyinosine	x	✓	x	x
2′-Deoxyadenosine	x	✓	✓	✓
Daidzein	x	✓	✓	✓
Glu-Gln	x	✓	✓	✓
Ile-Glu	x	✓	x	x
Tetradecanedioic acid	x	X	x	✓
Glu-Asn	✓	✓	x	x
Glu-asp	✓	✓	✓	✓
Gamma-Glutamylisoleucine	x	X	x	✓
Asp-Glu	x	X	✓	✓
Î^3^-Glutamylaspartic acid	x	✓	x	x
Methionylleucine	x	X	✓	x
Thiamine	✓	X	x	x
Inosine	✓	X	x	✓
Adenosine	✓	✓	✓	✓
Coumestrol	✓	✓	x	x
Genistein	✓	✓	✓	✓
Butein	✓	X	x	x
Palmitic acid	✓	X	x	x
3′-Hydroxy-O-desmethylangolensin	✓	X	x	x
Gln-Gln	✓	✓	✓	✓
12-Oxo phytodienoic acid	✓	✓	✓	✓
Gln-Glu	x	✓	✓	x
a-Linolenic acid	x	X	x	✓
Leu-Phe	x	✓	x	x
Linoleic acid	x	X	✓	x
Calycosin	✓	✓	✓	x
5-Deoxycajanin	x	X	x	✓
Ophthalmic acid	✓	✓	✓	x
Leu-Arg	x	✓	x	x
Catechin	x	✓	x	x
Gamma-Glutamyltyrosine	✓	✓	✓	✓
Argininosuccinic acid	✓	X	x	x
13(S)-HOTrE	x	X	x	✓
8-{3-Oxo-2-[(2E)−2-penten-1-yl]−1-cyclopenten-1-yl}octanoic acid	x	X	x	✓
13-OxoODE	✓	X	x	✓
Glycyl-prolyl-glutamic acid	✓	X	✓	✓
His-Glu	✓	X	x	x
(9Z.12Z)−7.8.16-trihydroxyoctadeca-9.12-dienoic acid	x	X	x	✓
Pro-Lys-Ser	x	X	x	✓
Glycerol monopalmitate	x	✓	x	x
Monopalmitin	✓	X	x	x
Isorhamnetin	x	✓	x	x
Leucyltryptophan	x	✓	x	x
Phytosphingosine	x	X	x	✓
Guanosine	✓	✓	✓	✓
4-Hydroxybenzoic acid glucoside	✓	✓	✓	x
2-Hydroxyadipic acid	x	X	x	✓
Cyclic AMP	✓	X	x	x
cIMP	✓	✓	x	x
1-(sn-glycero-3-phospho)−1D-myo-inositol	✓	X	x	✓
4-(beta-D-Glucopyranosyloxy)phenylacetic acid	x	X	✓	✓
1-Linoleoyl glycerol	✓	X	x	x
Asn-Ile-Leu	x	X	✓	✓
Sucrose	✓	✓	✓	✓
Cyclic GMP	✓	X	x	x
4-O-beta-D-glucosyl-4-coumaric acid	✓	✓	✓	✓
3-Methoxy-4-[3,4,5-trihydroxy-6-(hydroxymethyl)oxan-2-yl]oxybenzoic acid	x	X	✓	✓
Cellobiose	✓	X	x	x
3-[2-[3,4,5-Trihydroxy-6-(hydroxymethyl)oxan-2-yl]oxyphenyl]prop-2-enoic acid	x	X	x	✓
4′-O-beta-D-glucosyl-cis-p-coumaric acid	x	X	✓	x
5.7-Dihydroxy-6-[3,4,5-trihydroxy-6-(hydroxymethyl)oxan-2-yl]chromen-4-one	x	X	x	✓
3-Ketosucrose	✓	X	x	x
D-Maltose	✓	X	x	x
S-Adenosylmethionine	x	✓	x	x
Uridine 5′-diphosphate	✓	✓	x	x
Daidzein-4′-glucoside	x	✓	x	✓
Daidzin	✓	✓	x	x
Sucrose phosphate	✓	X	x	x
Asn-Thr-Tyr	✓	✓	✓	✓
Oleanolic acid	✓	X	✓	x
Gln-Leu-Tyr	x	X	x	✓
Luteolin 8-glucoside	✓	X	x	x
(-)-Epigallocatechin 3-(4-methyl-gallate)	x	X	x	✓
ACETYLGLYCITIN	x	✓	x	x
LysoPC(16:0/0:0)	✓	X	x	✓
Malonyldaidzin	✓	✓	✓	✓
Maltotriose	x	✓	x	✓
D-Raffinose pentahydrate	x	X	✓	x
Malonylgenistin	✓	✓	✓	✓
Cyclic ADP-ribose	x	X	✓	✓
Uridine 5′-diphosphogalactose	✓	X	x	x
UDP-GlcNAc	x	X	x	✓
UDP-N-acetylglucosamine	✓	X	✓	x
Soyasaponin A1	✓	X	x	x
(-)-Epigallocatechin	x	✓	x	x
Nicotinamide adenine dinucleotide (NAD+)	✓	✓	✓	x
FAD	✓	X	x	✓
PC(20:0/P-18:0)	x	X	✓	✓
Sulfoquinovosyl diglyceride	✓	X	x	x
Soyasapogenol B 3-O-[a-L-rhamnosyl-(1->4)-b-D-galactosyl-(1->4)-b-D-glucuronide]	✓	X	x	x
Indolelactic acid	✓	X	x	x
L-proline	✓	X	x	✓
ß-D-Glucopyranuronic acid	✓	X	x	✓
5(E),9(Z),12(Z)-OCTADECATRIENOIC ACID	✓	X	x	x
7-Hydroxy-2-(4-hydroxyphenyl)−4-oxo-3,4-dihydro-2H-chromen-5-yl ß-D-glucopyranoside	✓	X	x	x
5,6-Dihydrouridine	✓	X	x	x
3′,4′,7-Trihydroxyflavanone	x	✓	x	✓
3′,4′,5,7-Tetrahydroxyisoflavanone	x	✓	x	✓
Daidzoside	x	✓	x	x
2′-Deoxyadenosine	x	✓	x	✓
Trans-aconitic acid	x	✓	x	x
1-O-(4-Hydroxybenzoyl)-ß-D-glucopyranose	x	✓	x	x
1,3-dilinolenoylglycerol	x	X	✓	x
3-O-ß-D-galactosyl-sn-glycerol	x	X	✓	✓
Ethylmalonic acid	x	X	✓	x
Glucaric acid	x	X	✓	x
2-hydroxy-5-[3,4,5-trihydroxy-6-(hydroxymethyl)oxan-2-yl]oxybenzoic acid	x	X	✓	✓
3-Methoxy-4-[3,4,5-trihydroxy-6-(hydroxymethyl)oxan-2-yl]oxybenzoic acid	x	X	✓	✓
Phloroglucinol	x	X	✓	x
(6E,8E)−5,10-dioxooctadeca-6,8-dienoic acid	x	X	x	✓
1,16-Hexadecanedioic acid	x	X	X	✓
5,7-Dihydroxy-6-[3,4,5-trihydroxy-6-(hydroxymethyl)oxan-2-yl]chromen-4-one	x	X	X	✓

Calluses exposed to intermediate CuNP concentrations (10 and 50 µg L^−1^) showed marked changes in chemical composition, indicating that nanoparticle-induced stress affected multiple biochemical pathways. As discussed above, reduced chemical diversity was observed at these concentrations, with several metabolites detected in the control group no longer observed after CuNP exposure. For example, 2-isopropylmalic acid, LysoPC(16:0/0:0), 13-OxoODE, aminoadipic acid, and cyclic ADP-ribose, compounds associated with amino acid metabolism, nucleotide turnover, and stress signaling, were absent under these conditions. This reduction suggests an early metabolic suppression accompanied by reallocation of cellular resources, likely driven by mild oxidative stress and the redirection of energy toward maintaining redox balance and cellular homeostasis [28].

At 50 µg L^−1^, the detection of vanillic acid, coumaric acid, and several glycosylated phenolic compounds suggests activation of compensatory responses, potentially related to reinforcement of antioxidant capacity and stabilization of cellular redox status. This non-linear, dose-dependent response, where intermediate stress levels promote metabolic readjustment, is consistent with previous studies on plant-nanoparticle interactions [29–31].

In contrast, the exclusive detection of multiple metabolites in soybean calluses exposed to 100 µg L^−1^ CuNPs suggests coordinated perturbation of several interconnected pathways, indicating a transition from metabolic homeostasis toward stress-driven reprogramming.

One of the most strongly affected pathways was lipid metabolism, particularly oxylipin biosynthesis and fatty acid oxidation, as evidenced by the appearance of highly oxygenated fatty acid derivatives such as 13(S)-HOTrE, (6E,8E)−5,10-dioxooctadeca-6,8-dienoic acid, and (9Z,12Z)−7,8,16-trihydroxyoctadeca-9,12-dienoic acid [32, 33]. These metabolites are well-recognized markers of lipid peroxidation and membrane oxidation, suggesting that high CuNP exposure promotes a shift in lipid-derived signaling toward oxidative damage. Their absence in control calluses supports the interpretation that basal conditions do not generate sufficient oxidative pressure to trigger membrane lipid oxidation.

Although these findings strongly support stress-driven metabolic reprogramming, it cannot be excluded that part of the observed metabolic response reflects adaptive remodeling rather than irreversible cellular damage.

In parallel, redox homeostasis and glutathione-dependent metabolism appeared strongly perturbed at 100 µg L^−1^, as evidenced by the accumulation of metabolites associated with antioxidant turnover, including ophthalmic acid, γ-glutamylisoleucine, DL-pyroglutamic acid, dehydroascorbic acid, and S-methyl-L-cysteine-S-oxide [34]. Ophthalmic acid, in particular, is widely recognized as an indicator of glutathione depletion [35], suggesting that antioxidant buffering capacity may be exceeded under severe copper-induced stress.

Secondary metabolism derived from the phenylpropanoid and flavonoid pathways also emerged as a major stress-responsive route [36, 37]. This was supported by the exclusive detection of phenolic and flavonoid metabolites such as (–)-epigallocatechin 3-(4-methyl-gallate), 3′,4′,5,7-tetrahydroxyisoflavanone, daidzein, daidzein-4′-glucoside, licoflavone A, ginnalin B, and 4-hydroxycinnamic acid [38, 39]. These compounds likely contribute to antioxidant defense through radical scavenging and metal-chelating activities.

High CuNP exposure also reprogrammed amino acid metabolism, nitrogen remobilization, and the γ-aminobutyric acid (GABA) shunt [40], as indicated by the accumulation of GABA, D-α-aminobutyric acid, L-asparagine, L-threonine, and related metabolites. Additionally, the detection of ureide-related compounds such as allantoin, allantoic acid, and 2-ureidoglycine [41] suggests increased nitrogen storage and redistribution, a typical stress response in soybean.

Alterations in nucleotide metabolism and cofactor turnover were also evident, with exclusive detection of 1-methyladenine, 2′-deoxyadenosine, FAD, and UDP-GlcNAc, suggesting enhanced nucleotide recycling, possible DNA repair responses, and increased demand for redox-active cofactors [42–44].

Finally, high CuNP exposure affected membrane remodeling and carbohydrate metabolism associated with osmoprotection and carbon reallocation. This was reflected by the presence of membrane-associated species such as PC(20:0/P-18:0), phytosphingosine, and 3-O-β-D-galactosyl-sn-glycerol, together with carbohydrate-related metabolites including D-xylulose and maltotriose [45, 46].

Taken together, these findings indicate that exposure to 100 µg L^−1^ CuNPs triggers coordinated alterations in lipid metabolism, redox regulation, phenolic metabolism, amino acid and nitrogen metabolism, nucleotide turnover, and carbohydrate metabolism—pathways that remain comparatively stable under basal conditions.

**Fig. 5:**
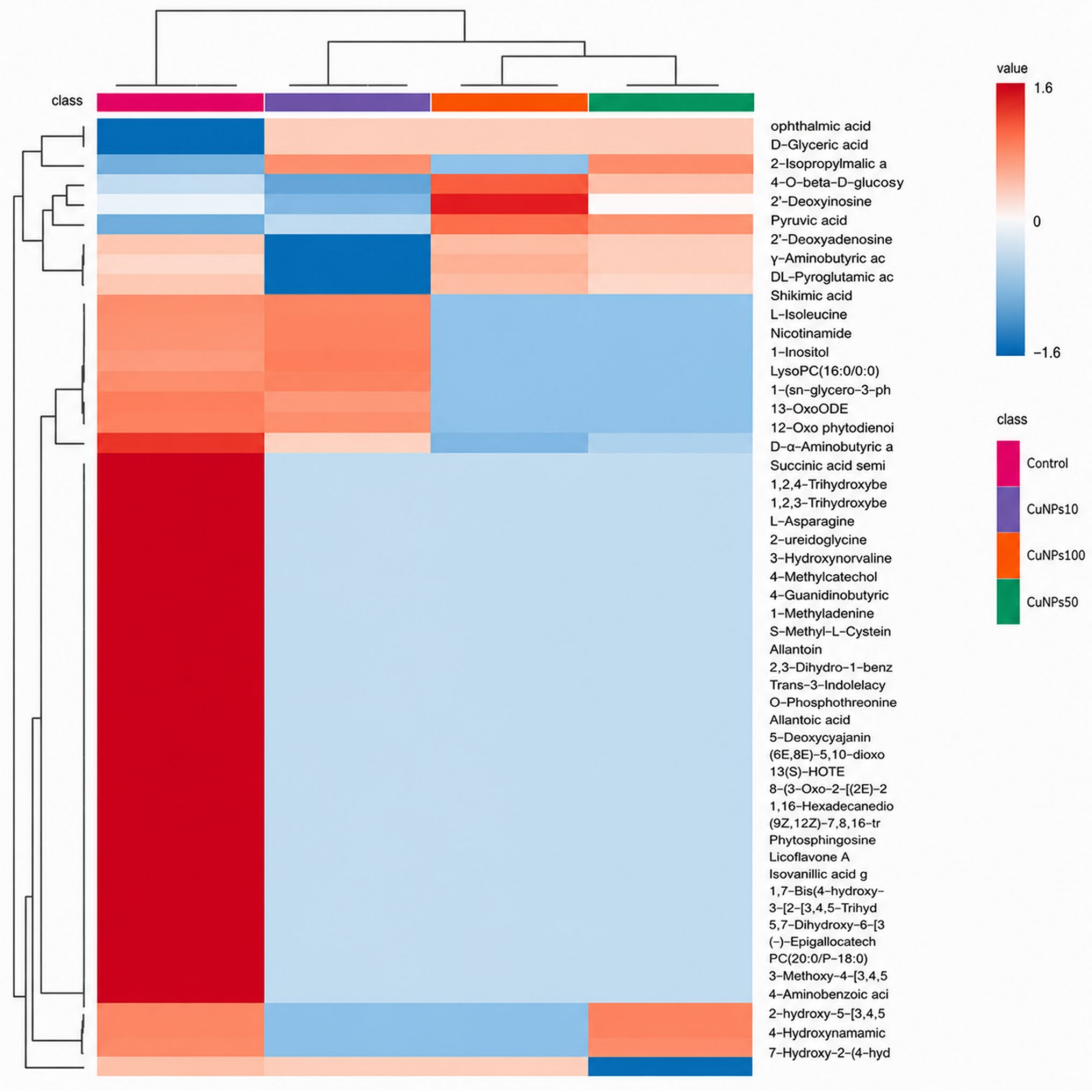
Heatmap of stress-responsive chemical species in transgenic soybean calluses exposed to different concentrations of CuNPs

To provide an integrated visualization of these stress-associated responses, a heatmap was constructed using selected metabolites associated with oxidative stress, redox imbalance, membrane remodeling, and secondary defense pathways (Fig. 5). The heatmap revealed clear concentration-dependent clustering and pronounced induction of stress-related metabolites at 100 µg L^−1^ CuNPs, whereas samples exposed to 10 and 50 µg L^−1^ exhibited lower abundance or suppression of these compounds, consistent with more adaptive metabolic states. These findings are consistent with recent integrated physiological, transcriptomic, and metabolomic studies showing that Cu-based nanoparticles can extensively remodel plant metabolic networks under stress conditions [47].

### Identification of known and putatively annotated heteroatom and metal-containing biomolecules in transgenic soybean calluses

The cultivation of soybean calluses exposed to CuNPs indicates that the cells undergo dynamic adjustments in both metal and non-metal homeostasis. These changes likely involve the formation and redistribution of diverse elemental complexes within the cellular environment, although detailed information regarding their chemical speciation in plant tissues remains limited. In soybean calluses, these elements may interact with a wide range of biomolecules, including metalloenzymes, metalloproteins, and low molecular weight ligands, thereby influencing numerous biological processes. Such interactions play essential roles in metabolic regulation, redox homeostasis, stress adaptation, and cellular development, highlighting the complexity of elemental dynamics in soybean calluses exposed to CuNPs [36, 37].

Table 3 summarizes the putatively annotated metal- and heteroatom-containing chemical species identified in transgenic soybean calluses cultured under 100 µg L^−1^ CuNPs. Detailed information regarding the species detected under the other experimental conditions is provided in Supplementary Tables S4–S6. Several amino acids, organic acids, phospholipids, and glycopeptide-like ligands were putatively associated with metal ions such as magnesium (Mg^2+^), manganese (Mn^2+^), and zinc (Zn^2+^), as well as with heteroatoms including sulfur (S) and phosphorus (P). These observations suggest that CuNP exposure may induce broader homeostatic adjustments involving redistribution of essential elements and their coordination with endogenous biomolecules.

**Table 3:** Putatively annotated chemical species identified in transgenic soybean calluses cultured at 100 µg L^−1^ of CuNPs using online integrated HILIC-ESI-HRMS-ICP-MS/MS

Chemical species	Annot. ∆Mass (ppm)	Calc. MW	m/z	Reference ion	RT (min)	Polarity	MS2 purity (%)
C29 H43 O4 P	−0.81	486.2895	485.2822	[M−H]−1	0.661	Negative	100
C32 H64 N8 O4 P2	−0.25	686.4524	685.4451	[M−H]−1	0.982	Negative	100
C37 H67 O10 P	0.13	702.4473	701.44	[M−H]−1	0.99	Negative	100
C24 H23 N O16	−0.08	581.1016	580.0944	[M−H]−1	1.046	Negative	100
C23 H30 Mn N2 O8 P2	0.07	579.0858	578.0786	[M−H]−1	1.05	Negative	100
C21 H22 N6 O4 P2 S	−0.5	516.0896	517.0969	[M+H]+1	1.054	Positive	100
C23 H32 Mn N2 O7 P2	−0.33	565.1063	564.0991	[M−H]−1	1.093	Negative	100
C15 H19 N4 O9 P	1.54	430.0896	429.0824	[M−H]−1	1.225	Both	100
C28 H24 O5 P2	0.82	502.1103	503.1176	[M+H]+1	1.475	Positive	100
C18 H21 N4 O12 P	0.56	516.0897	517.0969	[M+H]+1	1.58	Positive	100
C17 H14 N8 O12 S	0.13	554.0453	555.0526	[M+H]+1	1.581	Positive	100
C21 H16 O9	−1.09	412.079	471.0928	[M−H+HAc]−1	1.603	Negative	100
C19 H23 N4 O13 P	0.29	546.1001	547.1074	[M+H]+1	1.608	Positive	100
C24 H20 Mn N2 O6	−0.21	487.0701	486.0628	[M−H]−1	1.76	Negative	100
C15 H35 Mn N7 P2 S	−0.8	462.1527	463.1599	[M+H]+1	1.778	Positive	100
C19 H41 Mn N6 O15 P S	0.08	711.1469	710.1397	[M−H]−1	1.806	Negative	100
C29 H44 Mn N2 O8 P2 S	0.12	697.1675	696.1602	[M−H]−1	1.84	Negative	100
C13 H12 N4 O5	−4.94	304.0793	303.072	[M−H]−1	10.802	Negative	100
C12 H23 N O14	−0.74	405.1116	404.1043	[M−H]−1	13.221	Negative	100
C14 H14 N6 O3 S2	−2.77	378.0558	379.0631	[M+H]+1	13.298	Positive	100
C21 H35 N O6 P2	2.38	459.1951	458.1878	[M−H]−1	13.52	Negative	100
C7 H19 N7 O9	−2.63	345.1235	344.1162	[M−H]−1	14.408	Negative	100
C16 H25 N5 O9	−3.94	431.1635	430.1563	[M−H]−1	15.404	Negative	100
C8 H20 Mn N4 O7 S	2.31	371.0442	372.0515	[M+H]+1	16.747	Positive	100
C9 H19 N O8	0.12	269.1111	268.1038	[M−H]−1	16.866	Negative	100
C6 H19 N5 O3 S2	2.94	273.0937	272.0865	[M−H]−1	18.885	Negative	100
C9 H23 N4 O3 P S2	−2.76	330.094	329.0867	[M−H]−1	18.931	Negative	100
C17 H32 N2 O14	−0.45	488.1851	487.1779	[M−H]−1	19.021	Negative	100
C17 H30 N2 O14	−0.28	486.1696	485.1623	[M−H]−1	19.388	Negative	100
C12 H17 Mn N7 O2 S	3.55	378.0558	379.0631	[M+H]+1	19.404	Positive	100
C20 H28 N6 O8	−1.22	480.1963	481.2036	[M+H]+1	19.628	Positive	100
C18 H29 N3 O10	1.76	447.1861	448.1934	[M+H]+1	20.927	Positive	100
C19 H27 N O5 P2 S	2.04	443.1094	442.1023	[M−H]−1	21.369	Both	100
C3 H N2 O3 P S	0.25	175.9446	174.9373	[M−H]−1	23.451	Negative	100
C4 H3 N2 O5 P S2	−0.71	253.9219	252.9146	[M−H]−1	23.582	Negative	100
C20 H33 N O17	−0.63	559.1745	558.1674	[M−H]−1	26.628	Both	100
C12 H21 N O11	−0.54	355.1113	354.104	[M−H]−1	28.788	Both	100
C27 H38 Mn N2 O5 P2	−0.35	587.1634	588.1707	[M+H]+1	3.729	Positive	100
C26 H39 Mn N O6 P2	−0.31	578.1631	579.1704	[M+H]+1	4.168	Positive	100
C10 H11 N8 O2 P	−1.96	306.0737	635.1365	[2M+Na]+1	4.417	Positive	100
C20 H23 N O3 P2	2.66	387.1164	386.1091	[M−H]−1	4.889	Negative	100
C14 H14 N6 O3 S2	−2.77	378.0558	379.0631	[M+H]+1	6.03	Positive	100
C11 H23 N O13	−0.6	377.1167	376.1094	[M−H]−1	9.783	Negative	100
C11 H20 O6	−0.16	248.126	307.1397	[M−H+HAc]−1	2.717	Negative	91.93
C15 H11 Mg N3 O5	2.46	337.0557	338.063	[M+H]+1	1.232	Positive	92.61
C3 H8 Mn O6	−1.63	194.9698	195.9771	[M+H]+1	16.888	Positive	92.70
C10 H5 N5 O5	0.01	275.0291	293.0629	[M+NH4] 1	6.582	Positive	93.21
C11 H25 Mn N O3 S	3.65	306.0947	307.102	[M+H]+1	2.692	Positive	93.27
C4 H8 N8 O2	1.01	200.0772	199.07	[M−H]−1	16.639	Negative	94.58
C10 H20 N8 O6 S2	−1.01	412.0943	413.1015	[M+H]+1	2.082	Both	94.62
C10 H24 Mg N8 O3 P2	−3.48	390.1283	391.1356	[M+H]+1	2.442	Positive	94.68
C6 H11 Mn N O7	−1.3	263.9913	264.9985	[M+H]+1	7.452	Positive	94.79
C18 H29 N4 O5 P S2	0.64	476.132	475.1247	[M−H]−1	1.046	Negative	96.58
C22 H46 N7 O5 P	4.73	519.3323	520.3395	[M+H]+1	4.268	Positive	96.88
C13 H16 N4 O8	−3.94	356.0954	355.0881	[M−H]−1	23.818	Negative	97.12
C7 H3 N9	−0.03	213.0511	214.0584	[M+H]+1	21.405	Positive	97.33
C5 H10 N8 O3	0.52	230.0877	229.0804	[M−H]−1	16.976	Negative	97.43
C4 H3 N2 O5 P S2	−0.17	253.9221	252.9148	[M−H]−1	23.068	Negative	97.47
C16 H13 N6 O4 P	−3.81	384.0721	383.0649	[M−H]−1	3.84	Negative	97.48
C11 H22 O10	−0.64	314.1211	349.0905	[M+Cl]−1	9.803	Both	97.64
C16 H26 O3 S	−0.42	298.1601	297.1529	[M−H]−1	0.657	Negative	97.65
C7 H12 O7	−0.66	208.0582	207.0509	[M−H]−1	6.099	Negative	97.92
C22 H44 N7 O5 P	4.09	517.3163	518.3236	[M+H]+1	4.372	Positive	97.93
C6 H18 N5 O6 P	2.57	287.1002	288.1075	[M+H]+1	2.662	Positive	98.52
C17 H31 Mn N O6 S	2.92	432.1265	413.1088	[M−H−H2O]−1	2.734	Both	98.57
C7 H12 O8	−0.47	224.0531	223.0458	[M−H]−1	23.667	Negative	99.70

Notably, different metals exhibit distinct coordination preferences depending on ligand structure and chemical environment [17]. For example, the putative detection of a Mn-histidine complex suggests that CuNP-induced disruption of metal homeostasis may promote intracellular redistribution of manganese, potentially enabling its association with small biomolecules and the formation of organometallic-like complexes. Although these assignments remain putative, they provide valuable insights into possible elemental redistribution mechanisms triggered by nanoparticle exposure.

In general, the annotation of metal- and heteroatom-containing species was based on multiple complementary criteria, including accurate mass determination (< 5 ppm mass error), isotopic pattern matching, and MS/MS fragmentation analysis, combined with chromatographic co-elution between the elemental signal detected by ICP-MS/MS and the corresponding molecular ion detected by HRMS. Specifically, retention time agreement between the elemental/heteroatom trace and the associated molecular feature was verified for representative compounds, strengthening confidence in the proposed assignments [14, 17].

**Fig. 6:**
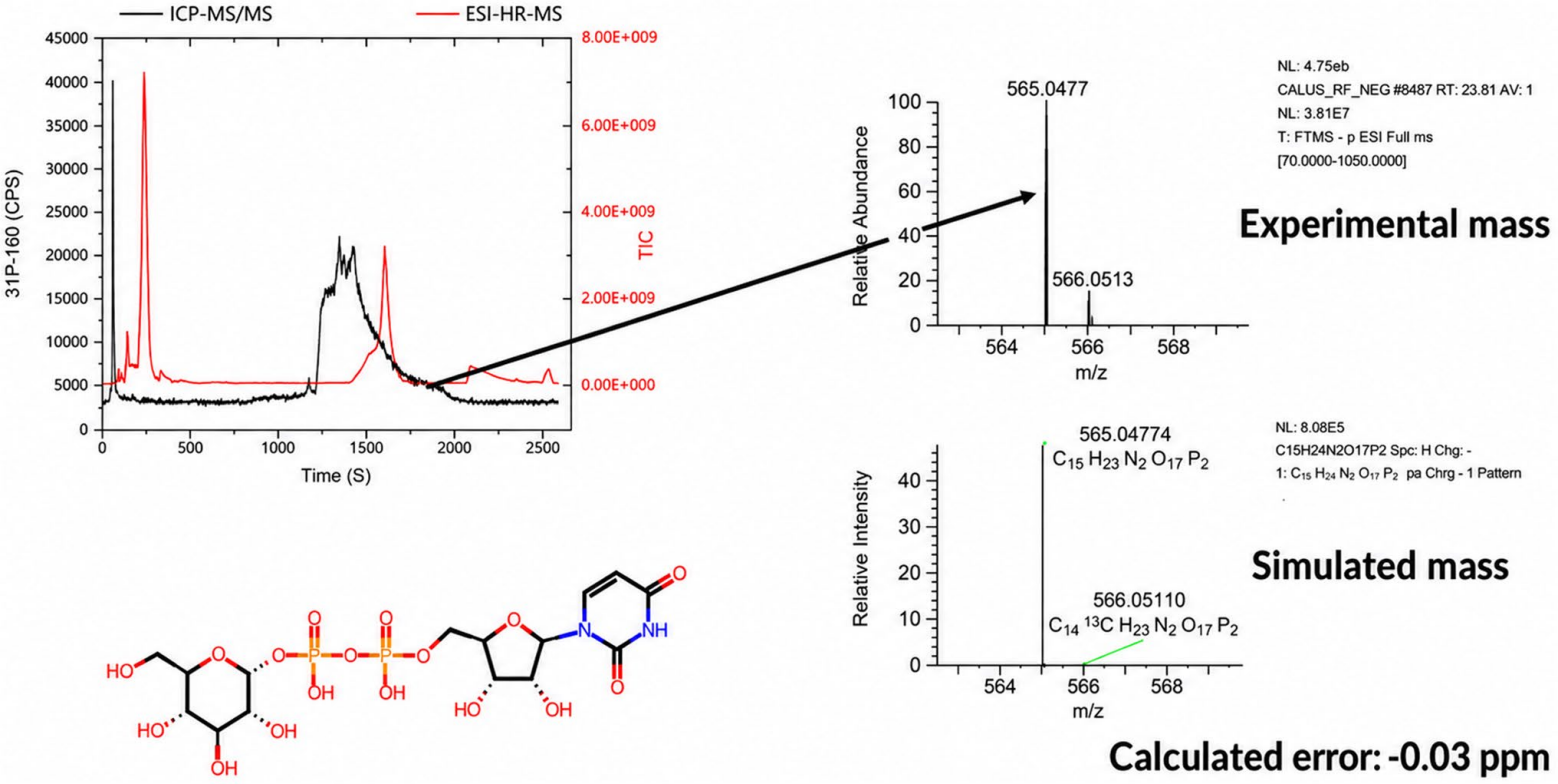
Example of the identification of the known P-containing metabolite uridine 5′-diphosphogalactose (C₁₅H₂₄N₂O₁₇P₂) in transgenic soybean calluses using HILIC-MS-ESI-HRMS-ICP-MS/MS

**Fig. 7:**
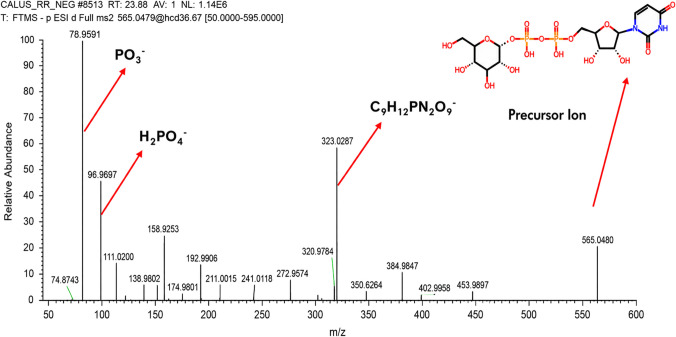
Fragmentation spectra for MS2 fragmentation, which shows the characteristics product ions used for structural confirmation of uridine 5′-diphosphogalactose (C₁₅H₂₄N₂O₁₇P₂)

Figure 6 illustrates a representative example of this workflow for the identification of the phosphorus-containing metabolite uridine 5′-diphosphogalactose (C15H24N2O17P2). Confirmation was based on accurate mass agreement (−0.03 ppm), isotopic distribution, and MS/MS fragmentation, including the precursor ion and characteristic product ions shown in Fig. 7, which support the proposed structural assignment. Additional representative examples of species containing Mg, Mn, S, and Zn are provided in Figs. S1–S4 in the Supplementary Material, demonstrating the applicability of this analytical strategy across different elemental classes.

Overall, these results reinforce the reliability of the integrated analytical workflow and demonstrate how online HILIC-ESI-HRMS-ICP-MS/MS can be used to annotate metal- and heteroatom-containing biomolecules in complex plant matrices. Beyond structural characterization, this approach provides important mechanistic insights into how elemental species participate in the cellular adjustments triggered by nanoparticle-induced stress.

## Conclusions

In this study, the integrated online HILIC-ESI-HRMS-ICP-MS/MS platform proved to be a robust and highly informative analytical strategy for the simultaneous molecular and elemental characterization of transgenic soybean calluses exposed to increasing concentrations of copper nanoparticles (CuNPs). The online coupling enabled the chromatographic co-detection of organic compounds together with metal- and heteroatom-containing species, providing complementary metabolomic and speciation information within a single analytical workflow.

Exposure to increasing CuNP concentrations resulted in a broader and more diverse chemical profile, particularly at the highest dose, where metabolites associated with cellular defense, oxidative stress, and membrane remodeling were markedly enriched. This concentration-dependent response highlights the capability of the integrated platform to capture stress-induced metabolic reprogramming that would be difficult to resolve using conventional single-detector approaches.

Importantly, the speciomics framework enabled by HILIC-ESI-HRMS-ICP-MS/MS allowed the putative annotation of metal-associated metabolites and heteroatom-containing complexes, revealing evidence of elemental redistribution involving essential elements and suggesting coordination-driven homeostatic adjustments at the cellular level.

From a biological perspective, the observed metabolic alterations indicate that CuNP exposure induces oxidative stress and triggers coordinated adaptive responses, including membrane remodeling, activation of antioxidant defense pathways, and reprogramming of amino acid and nitrogen metabolism. The accumulation of lipid-derived metabolites and oxylipins suggests enhanced lipid peroxidation and stress signaling, while the increased abundance of phenolic and related compounds reflects activation of protective biochemical mechanisms aimed at preserving cellular redox homeostasis.

Overall, this study advances the understanding of nanoparticle-plant interactions by demonstrating how CuNP exposure modulates both the molecular composition and elemental organization of soybean calluses. Beyond its biological relevance, this work highlights the analytical potential of integrated molecular and elemental coupling as a powerful platform for investigating complex biological systems and provides a valuable framework for future studies on nanoparticle-induced biochemical responses in the context of precision agriculture.

## Supplementary Information

Below is the link to the electronic supplementary material.

Supplementary file1 (DOCX 1.58 MB)

## Data Availability

All data generated and analyzed in this study are deposited in the Unicamp Research Data Repository (REDU).

## References

[1] Hazarika A, Yadav M, Yadav DK, Yadav HS. An overview of the role of nanoparticles in sustainable agriculture. Biocatal Agric Biotechnol. 2022;43:102399.

[2] Arruda MAZ, da Silva ABS, Kato LS. There is plenty of room in plant science: nanobiotechnology as an emerging area applied to somatic embryogenesis. J Agric Food Chem. 2023;71:3651–7. 36786777 10.1021/acs.jafc.2c08065

[3] Zheng X, Shou K, Hu C, Wu S, Sun J, Tan Q, Sun X. Molybdenum oxide nanoparticles improve soybean yield and enhance nutritional quality. J Food Compos Anal. 2024;132. 10.1016/j.jfca.2024.106307.

[4] de Campos BK, Galazzi RM, dos Santos BM, Balbuena TS, dos Santos FN, Mokochinski JB, Eberlin MN, Arruda MAZ. Comparison of generational effect on proteins and metabolites in non-transgenic and transgenic soybean seeds through the insertion of the cp4-EPSPS gene assessed by omics-based platforms. Ecotoxicol Environ Saf. 2020;202. 10.1016/j.ecoenv.2020.110918. 10.1016/j.ecoenv.2020.110918 32800253

[5] Hong HP, Zhang H, Olhoft P, Hill S, Wiley H, Toren E, et al. Organogenic callus as the target for plant regeneration and transformation via Agrobacterium in soybean (*Glycine max* (L.) Merr.). In Vitro Cell Dev Biol Plant. 2007;43:558–68. 10.1007/s11627-007-9066-1.

[6] da Silva ABS, Arruda MAZ. Single-cell ICP-MS to address the role of trace elements at a cellular level. J Trace Elem Med Biol. 2023;75. 10.1016/j.jtemb.2022.127086 36215757

[7] da Silva ABS, Arruda MAZ. Exploring single-particle ICP-MS as an important tool for the characterization and quantification of silver nanoparticles in a soybean cell culture. Spectrochim Acta Part B At Spectrosc. 2023;203. 10.1016/j.sab.2023.106663.

[8] Galazzi RM, Lopes Júnior CA, de Lima TB, Gozzo FC, Arruda MAZ. Evaluation of some effects on plant metabolism through proteins and enzymes in transgenic and non-transgenic soybeans after cultivation with silver nanoparticles. J Proteomics. 2019;191:88–106. 10.1016/j.jprot.2018.03.024 10.1016/j.jprot.2018.03.024 29581061

[9] Biswas P, Kumari A, Modi A, Priyam A, Haque R, Ola MS, Kumar S, Kumar N. Callus culture-derived regeneration and molecular characterization of regenerated Stevia rebaudiana: implications for steviol glycoside production and genetic stability. Front Plant Sci. 2025;16. 10.3389/fpls.2025.1566037. 10.3389/fpls.2025.1566037 PMC12408629 40918960

[10] Kroukamp EM, Wondimu T, Forbes PBC. Metal and metalloid speciation in plants: overview, instrumentation, approaches and commonly assessed elements. TrAC Trends Anal Chem. 2016;77:87–99.

[11] Feldmann J, Raab A, Krupp EM. Importance of ICPMS for speciation analysis is changing: future trends for targeted and non-targeted element speciation analysis. Anal Bioanal Chem. 2018;410:661–7. 10.1007/s00216-017-0502-8. 28735451 10.1007/s00216-017-0502-8 PMC5775347

[12] Chantada-Vázquez MP, Moreda-Piñeiro A, Barciela-Alonso MC, Bermejo-Barrera P. Spectrometric-based techniques for metal-binding protein assessment in clinical, environmental, and food samples. Appl Spectrosc Rev. 2017;52:145–74.

[13] Bluemlein K, Raab A, Feldmann J. Stability of arsenic peptides in plant extracts: off-line versus on-line parallel elemental and molecular mass spectrometric detection for liquid chromatographic separation. Anal Bioanal Chem. 2009;393:357–66. 10.1007/s00216-008-2395-z. 18821072 10.1007/s00216-008-2395-z

[14] Kato LS, Cerqueira da Silva VH, Campaci de Andrade D, Cruz G, Pedrobom JH, Raab A, Feldmann J, Arruda MAZ. Multimodal chemical speciation techniques based on simultaneous high resolution molecular/atomic mass spectrometry applied to online target/non-target analysis: A tutorial review. Anal Chim Acta. 2024;1331. 10.1016/j.aca.2024.343084 39532431

[15] Feldmann J, Salaün P, Lombi E. Critical review perspective: elemental speciation analysis methods in environmental chemistry-moving towards methodological integration. Environ Chem. 2009;6:275–89.

[16] Lorenc W, Hanć A, Sajnóg A, Barałkiewicz D. LC/ICP-MS and complementary techniques in bespoke and nontargeted speciation analysis of elements in food samples. Mass Spectrom Rev. 2022;41:32–50. 32997814 10.1002/mas.21662

[17] Flis P, Ouerdane L, Grillet L, Curie C, Mari S, Lobinski R. Inventory of metal complexes circulating in plant fluids: a reliable method based on HPLC coupled with dual elemental and high-resolution molecular mass spectrometric detection. New Phytol. 2016;211:1129–41. 10.1111/nph.13964. 27111838 10.1111/nph.13964

[18] Arruda MAZ, de Jesus JR, Blindauer CA, Stewart AJ. Speciomics as a concept involving chemical speciation and omics. J Proteomics. 2022;263. 10.1016/j.jprot.2022.104615 35595056

[19] Schmitt CR, da Silva Leal KN, Fonseca EKB, Kato LS, Arruda MAZ. Sustainable biogenic synthesis of copper nanoparticles using *Glycine max* calli and their circular role in callus regeneration. Anal Bioanal Chem. 2025;417:6707–17. 10.1007/s00216-025-06163-w. 41175219 10.1007/s00216-025-06163-w

[20] da Silva Ferreira D, da Silva Rodrigues L, Pereira FMV, Pereira-Filho ER. Principal component analysis (PCA) for chemical data evaluation and heat maps preparation: a tutorial. Quim Nova. 2023;46:747–754. 10.21577/0100-4042.20230030.

[21] Xu E, Liu Y, Gu D, Zhan X, Li J, Zhou K, et al. Molecular mechanisms of plant responses to copper: from deficiency to excess. Int J Mol Sci. 2024. 10.3390/ijms25136993. 39000099 10.3390/ijms25136993 PMC11240974

[22] Sugimoto K, Allmann S, Kolomiets MV. Editorial: oxylipins: the front line of plant interactions. Front Plant Sci. 2022. 10.3389/fpls.2022.878765. 35419016 10.3389/fpls.2022.878765 PMC9000970

[23] Khan N. Exploring plant resilience through secondary metabolite profiling: advances in stress response and crop improvement. Plant Cell Environ. 2025;48:4823–37. 40091600 10.1111/pce.15473

[24] Shi B, Ding H, Wang L, Wang C, Tian X, Fu Z, et al. Investigation on the stability in plant metabolomics with a special focus on freeze-thaw cycles: LC–MS and NMR analysis to Cassiae Semen (*Cassia obtusifolia* L.) seeds as a case study. J Pharm Biomed Anal. 2021. 10.1016/j.jpba.2021.114243. 34273658 10.1016/j.jpba.2021.114243

[25] Coutinho ID, Henning LMM, Döpp SA, Nepomuceno A, Moraes LAC, Marcolino-Gomes J, et al. Identification of primary and secondary metabolites and transcriptome profile of soybean tissues during different stages of hypoxia. Data Brief. 2018;21:1089–100. 10.1016/j.dib.2018.09.122. 30450404 10.1016/j.dib.2018.09.122 PMC6226558

[26] Silvente S, Sobolev AP, Lara M. Metabolite adjustments in drought tolerant and sensitive soybean genotypes in response to water stress. PLoS One. 2012. 10.1371/journal.pone.0038554. 22685583 10.1371/journal.pone.0038554 PMC3369847

[27] Quintela AL, Santos MFC, de Lima RF, Mayer JLS, Marcheafave GG, Arruda MAZ, et al. Influence of silver nanoparticles on the metabolites of two transgenic soybean varieties: an NMR-based metabolomics approach. J Agric Food Chem. 2024;72:12281–94. 10.1021/acs.jafc.4c00756. 38747520 10.1021/acs.jafc.4c00756 PMC11140748

[28] Xu Y, Freund DM, Hegeman AD, Cohen JD. Metabolic signatures of *Arabidopsis thaliana* abiotic stress responses elucidate patterns in stress priming, acclimation, and recovery. Stress Biol. 2022. 10.1007/s44154-022-00034-5. 37676384 10.1007/s44154-022-00034-5 PMC10441859

[29] Sharma P, Chauhan N. Molecular mechanisms of nanomaterial interaction with plants. In: Nanotechnology for abiotic stress tolerance and management in crop plants. Elsevier; 2024. p. 77–93.

[30] Majumdar S, Long RW, Kirkwood JS, Minakova AS, Keller AA. Unraveling metabolic and proteomic features in soybean plants in response to copper hydroxide nanowires compared to a commercial fertilizer. Environ Sci Technol. 2021;55:13477–89. 10.1021/acs.est.1c00839. 34240865 10.1021/acs.est.1c00839

[31] Guo Y, Li H, Hao Y, Shang H, Jia W, Liang A, et al. Size effects of copper oxide nanoparticles on boosting soybean growth via differentially modulating nitrogen assimilation. Nanomaterials. 2024. 10.3390/nano14090746. 38727340 10.3390/nano14090746 PMC11085672

[32] Knieper M, Viehhauser A, Dietz KJ. Oxylipins and reactive carbonyls as regulators of the plant redox and reactive oxygen species network under stress. Antioxid. 2023. 10.3390/antiox12040814. 10.3390/antiox12040814 PMC10135161 37107189

[33] Henschel JM, Andrade AN de, dos Santos JBL, da Silva RR, da Mata DA, Souza T, Batista DS. Lipidomics in plants under abiotic stress conditions: an overview. Agron. 2024;14.

[34] Foyer CH, Noctor G. Ascorbate and glutathione: the heart of the redox hub. Plant Physiol. 2011;155:2–18. 21205630 10.1104/pp.110.167569 PMC3075780

[35] Servillo L, Castaldo D, Giovane A, Casale R, D’Onofrio N, Cautela D, et al. Ophthalmic acid is a marker of oxidative stress in plants as in animals. Biochim Biophys Acta Gen Subj. 2018;1862:991–8. 10.1016/j.bbagen.2018.01.015. 29413907 10.1016/j.bbagen.2018.01.015

[36] Wu D, Saleem M, He T, He G. The mechanism of metal homeostasis in plants: a new view on the synergistic regulation pathway of membrane proteins, lipids and metal ions. Membranes. 2021. 10.3390/membranes11120984. 34940485 10.3390/membranes11120984 PMC8706360

[37] Mydy LS, Chigumba DN, Kersten RD. Plant copper metalloenzymes as prospects for new metabolism involving aromatic compounds. Front Plant Sci. 2021. 10.3389/fpls.2021.692108. 34925392 10.3389/fpls.2021.692108 PMC8672867

[38] Jańczak-Pieniążek M, Cichoński J, Michalik P, Chrzanowski G. Effect of heavy metal stress on phenolic compounds accumulation in winter wheat plants. Mol. 2023;28. 10.3390/molecules28010241. 10.3390/molecules28010241 PMC9822316 36615433

[39] Sharma A, Shahzad B, Rehman A, Bhardwaj R, Landi M, Zheng B. Response of phenylpropanoid pathway and the role of polyphenols in plants under abiotic stress. Mol. 2019. 10.3390/molecules24132452. 10.3390/molecules24132452 PMC6651195 31277395

[40] Al-Quraan NA, Al-Ajlouni ZI, Qawasma NF. Physiological and biochemical characterization of the GABA shunt pathway in pea (*Pisum sativum* L.) seedlings under drought stress. Horticulturae. 2021;7:060125. 10.3390/horticulturae7060125.

[41] Ono Y, Fukasawa M, Sueyoshi K, Ohtake N, Sato T, Tanabata S, et al. Application of nitrate, ammonium, or urea changes the concentrations of ureides, urea, amino acids and other metabolites in xylem sap and in the organs of soybean plants (*Glycine max* (L.) Merr.). Int J Mol Sci. 2021;22:094573. 10.3390/ijms22094573. 10.3390/ijms22094573 PMC8123890 33925462

[42] Chen YH, Cheng WH. Hexosamine biosynthesis and related pathways, protein N-glycosylation and O-GlcNAcylation: their interconnection and role in plants. Front Plant Sci. 2024. 10.3389/fpls.2024.1349064. 38510444 10.3389/fpls.2024.1349064 PMC10951099

[43] Mosa KA, El-Naggar M, Ramamoorthy K, Alawadhi H, Elnaggar A, Wartanian S, et al. Copper nanoparticles induced genotoxicty, oxidative stress, and changes in superoxide dismutase (SOD) gene expression in cucumber (*Cucumis sativus*) plants. Front Plant Sci. 2018. 10.3389/fpls.2018.00872. 30061904 10.3389/fpls.2018.00872 PMC6055047

[44] Lynch JH, Roje S. A higher plant FAD synthetase is fused to an inactivated FAD pyrophosphatase. J Biol Chem. 2022. 10.1016/j.jbc.2022.102626. 36273586 10.1016/j.jbc.2022.102626 PMC9678776

[45] Mashabela MD, Masamba P, Kappo AP. Applications of metabolomics for the elucidation of abiotic stress tolerance in plants: a special focus on osmotic stress and heavy metal toxicity. Plants. 2023. 10.3390/plants12020269. 36678982 10.3390/plants12020269 PMC9860948

[46] Silva S, Dias MC, Pinto DCGA, Silva AMS. Metabolomics as a tool to understand nano-plant interactions: the case study of metal-based nanoparticles. Plants. 2023. 10.3390/plants12030491. 36771576 10.3390/plants12030491 PMC9921902

[47] Tian H, Deng Y, Liao K, Xu S, Chen J, He L. Physiological, transcriptomic, and metabolomic analyses reveal the adaptation mechanism of *Betaphycus gelatinus* under different salinity conditions. Algal Res. 2025. 10.1016/j.algal.2025.103894.

