# Supplementary material for: Integrated online HILIC-ESI-HRMS and ICP-MS/MS for chemical species profiling in transgenic soybean callus exposed to copper nanoparticles
Source: Anal Bioanal Chem. 2026 Jun 13;418(15):4929–50. doi: 10.1007/s00216-026-06596-x (PMC13388509; doi:10.1007/s00216-026-06596-x)
Supplement: Supplementary file 1 — Supplementary file1 (DOCX 1.58 MB) [file 216_2026_6596_MOESM1_ESM.docx]

**Integrated online HILIC-ESI-HRMS and ICP-MS/MS for chemical species profiling in transgenic soybean callus exposed to copper nanoparticles**

Raimundo Rafael Gamela^1.2*^. Elisânia Kelly Barbosa Fonseca^1.2^. Vinnícius Henrique Cerqueira da Silva^1.2^. Cristiane Renata Schmitt^1.2^ and Marco Aurélio Zezzi Arruda^1.2^

^1^Spectrometry. Sample Preparation and Mechanization Group. Institute of Chemistry. Institute of Chemistry. University of Campinas (Unicamp). Campinas. São Paulo. 13083-970. Brazil.

^2^National Institute of Science and Technology in Bioanalytics – Lauro Kubota (INCTBio-LK). Institute of Chemistry. University of Campinas– Unicamp. P.O. Box 6154. Campinas. SP 13083-970. Brazil.

^*^Corresponding author: Raimundo Rafael Gamela

E-mail: ragamela@gmail.com

**SUPPLEMENTARY MATERIAL**

**Table captions**

**Table 1S.** Chemical species identified in transgenic soybean calluses under control conditions (absence of CuNPs) using online integrated HILIC-ESI-HRMS-ICP-MS/MS.

**Table 2S.** Chemical species identified in transgenic soybean calluses cultured at
10 µg L^-1^  of CuNPs using online integrated HILIC-ESI-HRMS-ICP-MS/MS.

**Table 3S.** Chemical species identified in transgenic soybean calluses cultured at 50 µg L^-1^ of CuNPs using online integrated HILIC-ESI-HRMS-ICP-MS/MS.

**Table 4S.** Putatively annotated heteroatoms and metal containing biomolecules identified in transgenic soybean calluses under control conditions (absence of CuNPs) using online integrated HILIC-ESI-HRMS-ICP-MS/MS.

**Table 5S.** Putatively annotated heteroatoms and metal containing biomolecules identified in transgenic soybean calluses exposed at 10 µg L^-1^ of CuNPs using online integrated HILIC-ESI-HRMS-ICP-MS/MS.

**Table 6S**. Putatively annotated heteroatoms and metal containing biomolecules identified in transgenic soybean calluses at 50 µg L^-1^ of CuNPs using online integrated HILIC-ESI-HRMS-ICP-MS/MS.

**Table 1S**

| **Chemical Species** | **Formulae** | **Annot. ∆Mass [ppm]** | **Calc.**  **MW** | **m/z** | **Reference**  **Ion** | **RT [min]** |
| --- | --- | --- | --- | --- | --- | --- |
| Indole | C8 H7 N | -0.27 | 117.0578 | 118.0651 | [M+H]+1 | 0.99 |
| Indolelactic acid | C11 H11 N O3 | -0.71 | 205.0738 | 206.0810 | [M+H]+1 | 6.91 |
| y-Aminobutyric acid (GABA) | C4 H9 N O2 | 1.12 | 103.0634 | 102.0562 | [M-H]-1 | 19.81 |
| D-Ribose | C5 H10 O5 | 0.35 | 150.0529 | 131.0350 | [M-H-H2O]-1 | 16.65 |
| Ala-Ala-Ala | C9 H17 N3 O4 | -1.06 | 231.1217 | 232.1289 | [M+H]+1 | 24.39 |
| Ala-Asp | C7 H12 N2 O5 | 0.89 | 204.0748 | 185.0570 | [M-H-H2O]-1 | 7.09 |
| Aminoadipic acid | C6 H11 N O4 | -0.27 | 161.0688 | 144.0655 | [M+H-H2O]+1 | 3.73 |
| Argininosuccinic acid | C10 H18 N4 O6 | -0.94 | 290.1224 | 291.1296 | [M+H]+1 | 22.30 |
| Betaine | C5 H11 N O2 | -0.11 | 117.0790 | 118.0862 | [M+H]+1 | 9.23 |
| D-Asparagine | C4 H8 N2 O3 | 0.37 | 132.0535 | 131.0463 | [M-H]-1 | 8.44 |
| Arginine | C6 H14 N4 O2 | 0.21 | 174.1117 | 175.1190 | [M+H]+1 | 1.10 |
| L-Glutamine | C5 H10 N2 O3 | -0.33 | 146.0691 | 147.0764 | [M+H]+1 | 22.92 |
| L-Threonine | C4 H9 N O3 | 1.26 | 119.0584 | 118.0511 | [M-H]-1 | 18.80 |
| Gamma-Glutamyltyrosine | C14 H18 N2 O6 | 1.04 | 310.1168 | 291.0990 | [M-H-H2O]-1 | 2.61 |
| Gln-Gln | C10 H18 N4 O5 | -1.06 | 274.1274 | 275.1347 | [M+H]+1 | 24.02 |
| Glu-Ala | C8 H14 N2 O5 | -0.83 | 218.0901 | 219.0974 | [M+H]+1 | 22.93 |
| Glu-Asn | C9 H15 N3 O6 | 0.58 | 261.0962 | 260.0890 | [M-H]-1 | 24.93 |
| Glu-asp | C9 H14 N2 O7 | 1.01 | 262.0804 | 261.0731 | [M-H]-1 | 23.24 |
| Glu-Gly | C7 H12 N2 O5 | -0.92 | 204.0744 | 205.0817 | [M+H]+1 | 22.96 |
| Glycyl-prolyl-glutamic acid | C12 H19 N3 O6 | -0.57 | 301.1272 | 302.1345 | [M+H]+1 | 6.83 |
| His-Glu | C11 H16 N4 O5 | -5.38 | 284.1105 | 307.0998 | [M+Na]+1 | 9.01 |
| Ile-Asp | C10 H18 N2 O5 | -0.03 | 246.1216 | 245.1143 | [M-H]-1 | 20.68 |
| L-Arginine | C6 H14 N4 O2 | -0.3 | 174.1116 | 197.1009 | [M+Na]+1 | 18.07 |
| L-Aspartic acid | C4 H7 N O4 | 0.32 | 133.0376 | 132.0303 | [M-H]-1 | 22.63 |
| L-Histidine | C6 H9 N3 O2 | -0.7 | 155.0694 | 156.0766 | [M+H]+1 | 19.62 |
| L-proline | C5 H9 N O2 | 0.21 | 115.0634 | 116.0706 | [M+H]+1 | 23.19 |
| L-Pyroglutamic acid | C5 H7 N O3 | 0.21 | 129.0426 | 130.0499 | [M+H]+1 | 7.49 |
| N6-Acetyl-L-lysine | C8 H16 N2 O3 | 1.68 | 188.1164 | 187.1091 | [M-H]-1 | 15.78 |
| N-Acetylglutamic acid | C7 H11 N O5 | 0.87 | 189.0639 | 188.0566 | [M-H]-1 | 13.79 |
| N-Acetylglycine | C4 H7 N O3 | 1.16 | 117.0427 | 116.0355 | [M-H]-1 | 5.15 |
| N-Acetyl-L-asparagine | C6 H10 N2 O4 | 0.24 | 174.0641 | 173.0568 | [M-H]-1 | 8.44 |
| N-Acetyl-L-aspartic acid | C6 H9 N O5 | 0.49 | 175.0482 | 174.0409 | [M-H]-1 | 10.28 |
| N-Acetyl-L-histidine | C8 H11 N3 O3 | 0.33 | 197.0801 | 196.0728 | [M-H]-1 | 15.80 |
| N-Acetyl-L-phenylalanine | C11 H13 N O3 | 0.34 | 207.0896 | 206.0823 | [M-H]-1 | 7.02 |
| N-acetylornithine | C7 H14 N2 O3 | 0.46 | 174.1005 | 173.0933 | [M-H]-1 | 22.43 |
| O-Acetylserine | C5 H9 N O4 | 0.43 | 147.0532 | 146.0459 | [M-H]-1 | 5.14 |
| Ophthalmic acid | C11 H19 N3 O6 | 0.35 | 289.1275 | 288.1202 | [M-H]-1 | 23.29 |
| O-Succinyl-L-homoserine | C8 H13 N O6 | -0.11 | 219.0743 | 218.0670 | [M-H]-1 | 10.51 |
| Val-Asp | C9 H16 N2 O5 | 0.15 | 232.1060 | 231.0987 | [M-H]-1 | 22.62 |
| Val-Glu | C10 H18 N2 O5 | 0.16 | 246.1216 | 245.1143 | [M-H]-1 | 22.61 |
| 3-Ketosucrose | C12 H20 O11 | -0.27 | 340.1005 | 375.0699 | [M+Cl]-1 | 19.51 |
| Cellobiose | C12 H22 O11 | 0.02 | 342.1162 | 360.1499 | [M+NH4]+1 | 21.12 |
| D-Fructose | C6 H12 O6 | 0.11 | 180.0634 | 215.0328 | [M+Cl]-1 | 4.03 |
| D-Raffinose pentahydrate | C18 H32 O16 | 0.25 | 504.1692 | 539.1384 | [M+Cl]-1 | 21.38 |
| D-Maltose | C12 H22 O11 | -0.12 | 342.1162 | 377.0856 | [M+Cl]-1 | 19.18 |
| Galactitol | C6 H14 O6 | -0.17 | 182.0790 | 181.0718 | [M-H]-1 | 5.55 |
| I-Inositol | C6 H12 O6 | 0.6 | 180.0635 | 179.0562 | [M-H]-1 | 16.99 |
| Phosphohexose | C6 H11 O8 P | -0.09 | 242.0191 | 241.0119 | [M-H]-1 | 19.69 |
| ß-D-Glucopyranuronic acid | C6 H10 O7 | 0.44 | 194.0427 | 193.0355 | [M-H]-1 | 17.62 |
| Sucrose | C12 H22 O11 | 0.29 | 342.1163 | 341.1090 | [M-H]-1 | 23.27 |
| Sucrose phosphate | C12 H23 O14 P | -0.45 | 422.0824 | 423.0896 | [M+H]+1 | 4.52 |
| Gluconolactone | C6 H10 O6 | 0.63 | 178.0479 | 177.0406 | [M-H]-1 | 5.54 |
| 12-Oxo phytodienoic acid | C18 H28 O3 | -0.98 | 292.2036 | 275.2003 | [M+H-H2O]+1 | 0.92 |
| 13-OxoODE | C18 H30 O3 | -1.75 | 294.2190 | 295.2263 | [M+H]+1 | 0.94 |
| Monopalmitin | C19 H38 O4 | -0.77 | 330.2768 | 313.2735 | [M+H-H2O]+1 | 0.76 |
| Palmitic acid | C16 H32 O2 | -0.15 | 256.2402 | 274.2740 | [M+NH4]+1 | 1.03 |
| 7-Hydroxy-2-(4-hydroxyphenyl)-4-oxo-3.4-dihydro-2H-chromen-5-yl ß-D-glucopyranoside | C21 H22 O10 | 0.41 | 434.1215 | 433.1142 | [M-H]-1 | 1.28 |
| Calycosin | C16 H12 O5 | 0.61 | 284.0687 | 283.0614 | [M-H]-1 | 1.17 |
| Coumestrol | C15 H8 O5 | -0.17 | 268.0371 | 269.0444 | [M+H]+1 | 1.09 |
| Daidzin | C21 H20 O9 | -0.65 | 416.1105 | 417.1177 | [M+H]+1 | 1.04 |
| Genistein | C15 H10 O5 | -1.06 | 270.0525 | 271.0598 | [M+H]+1 | 0.80 |
| Malonyldaidzin | C24 H22 O12 | -0.45 | 502.1109 | 503.1182 | [M+H]+1 | 1.16 |
| Malonylgenistin | C24 H22 O13 | 0.17 | 518.1061 | 519.1134 | [M+H]+1 | 2.36 |
| Sulfoquinovosyl diglyceride | C45 H76 O12 S | -0.29 | 840.5055 | 839.4982 | [M-H]-1 | 0.71 |
| 2'-Deoxyinosine | C10 H12 N4 O4 | -5.13 | 252.0846 | 251.0773 | [M-H]-1 | 9.17 |
| 5.6-Dihydrouridine | C9 H14 N2 O6 | 0.01 | 246.0852 | 245.0779 | [M-H]-1 | 11.01 |
| Adenosine | C10 H13 N5 O4 | 0.13 | 267.0968 | 268.1041 | [M+H]+1 | 1.73 |
| Cytidine | C9 H13 N3 O5 | 0.83 | 243.0857 | 242.0785 | [M-H]-1 | 3.16 |
| Guanosine | C10 H13 N5 O5 | -0.92 | 283.0914 | 322.0546 | [M+K]+1 | 3.91 |
| Inosine | C10 H12 N4 O5 | 0.66 | 268.0810 | 267.0737 | [M-H]-1 | 2.51 |
| Pseudouridine | C9 H12 N2 O6 | -0.2 | 244.0695 | 243.0622 | [M-H]-1 | 3.28 |
| Uridine | C9 H12 N2 O6 | 1.47 | 244.0699 | 243.0628 | [M-H]-1 | 1.68 |
| cIMP | C10 H11 N4 O7 P | -3.58 | 330.0354 | 329.0281 | [M-H]-1 | 21.97 |
| Cyclic ADP-ribose | C15 H21 N5 O13 P2 | 0.12 | 541.0612 | 540.0539 | [M-H]-1 | 23.99 |
| Cyclic AMP | C10 H12 N5 O6 P | 0.79 | 329.0528 | 328.0455 | [M-H]-1 | 2.80 |
| Cyclic GMP | C10 H12 N5 O7 P | 0.19 | 345.0475 | 344.0402 | [M-H]-1 | 8.55 |
| Nicotinamide | C6 H6 N2 O | 0.19 | 122.0480 | 123.0553 | [M+H]+1 | 18.17 |
| Nicotinamide adenine dinucleotide (NAD+) | C21 H27 N7 O14 P2 | -0.19 | 663.1090 | 664.1163 | [M+H]+1 | 23.95 |
| UDP-N-acetylglucosamine | C17 H27 N3 O17 P2 | 0.41 | 607.0818 | 606.0745 | [M-H]-1 | 22.65 |
| Uridine 5'-diphosphate | C9 H14 N2 O12 P2 | -0.48 | 404.0020 | 405.0093 | [M+H]+1 | 23.76 |
| Uridine 5'-diphosphogalactose | C15 H24 N2 O17 P2 | -0.03 | 566.0550 | 565.0477 | [M-H]-1 | 23.82 |
| 2-Isopropylmalic acid | C7 H12 O5 | -0.22 | 176.0684 | 175.0612 | [M-H]-1 | 8.30 |
| Ascorbic acid | C6 H8 O6 | 0.69 | 176.0322 | 175.0249 | [M-H]-1 | 16.61 |
| Citric acid | C6 H8 O7 | 0.97 | 192.0272 | 191.0199 | [M-H]-1 | 23.24 |
| D-Glyceric acid | C3 H6 O4 | 0.58 | 106.0267 | 105.0194 | [M-H]-1 | 5.55 |
| Lactic Acid | C3 H6 O3 | 0.81 | 90.0318 | 89.0245 | [M-H]-1 | 2.49 |
| D-Pantothenic acid | C9 H17 N O5 | 0.41 | 219.1108 | 218.1035 | [M-H]-1 | 2.49 |
| Pyruvic acid | C3 H4 O3 | 0.68 | 88.0161 | 87.0088 | [M-H]-1 | 3.98 |
| Cis-Aconitic acid | C6 H6 O6 | 0.58 | 174.0165 | 173.0093 | [M-H]-1 | 2.60 |
| Gluconic acid | C6 H12 O7 | 0.39 | 196.0584 | 195.0511 | [M-H]-1 | 13.35 |
| 1-(sn-glycero-3-phospho)-1D-myo-inositol | C9 H19 O11 P | 0.31 | 334.0666 | 333.0593 | [M-H]-1 | 22.64 |
| Glycerophosphoglycerol | C6 H15 O8 P | -0.16 | 246.0504 | 245.0431 | [M-H]-1 | 11.95 |
| LysoPC(16:0/0:0) | C24 H50 N O7 P | 1 | 495.3330 | 496.3394 | [M+H]+1 | 4.54 |
| Adenine | C5 H5 N5 | 0.08 | 135.0545 | 136.0618 | [M+H]+1 | 1.57 |
| Guanine | C5 H5 N5 O | 0.18 | 151.0494 | 150.0422 | [M-H]-1 | 2.79 |
| Cytosine | C4 H5 N3 O | 0.19 | 111.0433 | 112.0506 | [M+H]+1 | 3.17 |
| Soyasaponin A1 | C59 H96 O29 | -1.3 | 1268.6021 | 633.2938 | [M-2H]-2 | 17.97 |
| Soyasapogenol B 3-O-[a-L-rhamnosyl-(1->4)-b-D-galactosyl-(1->4)-b-D-glucuronide] | C48 H78 O18 | -0.23 | 942.5186 | 981.4819 | [M+K]+1 | 4.40 |
| Butein | C15 H12 O5 | -0.69 | 272.0683 | 273.0756 | [M+H]+1 | 4.52 |
| Protocatehuic acid | C7 H6 O4 | 0.5 | 154.0267 | 153.0194 | [M-H]-1 | 8.55 |
| Shikimic acid | C7 H10 O5 | 0.53 | 174.0529 | 173.0456 | [M-H]-1 | 1.27 |
| Mevalonic acid | C6 H12 O4 | 0.84 | 148.0737 | 147.0664 | [M-H]-1 | 2.34 |
| Oleanolic acid | C30 H48 O3 | 0.58 | 456.3606 | 455.3533 | [M-H]-1 | 0.77 |
| Pyridoxal | C8 H9 N O3 | -0.27 | 167.0582 | 150.0549 | [M+H-H2O]+1 | 1.33 |
| Thiamine | C12 H16 N4 O S | -0.82 | 264.1043 | 265.1115 | [M+H]+1 | 20.52 |

**Table 2S**

| **Chemical species** | **Formulae** | **Annot. ∆Mass [ppm]** | **Calc. MW** | **m/z** | **Reference**  **Ion** | **RT**  **[min]** |
| --- | --- | --- | --- | --- | --- | --- |
| Indole | C8 H7 N | -0.21 | 117.0578 | 118.0651 | [M+H]+1 | 1.06 |
| Trigonelline | C7 H7 N O2 | -0.17 | 137.0477 | 138.0549 | [M+H]+1 | 9.81 |
| y-Aminobutyric acid (GABA) | C4 H9 N O2 | -0.04 | 103.0633 | 104.0706 | [M+H]+1 | 19.73 |
| y-Glu-Ala | C8 H14 N2 O5 | 0.34 | 218.0904 | 217.0831 | [M-H]-1 | 25.19 |
| Ala-Ile | C9 H18 N2 O3 | 0.07 | 202.1318 | 201.1246 | [M-H]-1 | 10.40 |
| Asn-Thr-Tyr | C17 H24 N4 O7 | -4.45 | 396.1627 | 435.1259 | [M+K]+1 | 2.59 |
| Citrulline | C6 H13 N3 O3 | -0.4 | 175.0956 | 159.0764 | [M+H-NH3]+1 | 21.32 |
| L-Glutamine | C5 H10 N2 O3 | -0.23 | 146.0691 | 147.0764 | [M+H]+1 | 25.03 |
| DL-Pyroglutamic acid | C5 H7 N O3 | 0.25 | 129.0426 | 130.0499 | [M+H]+1 | 18.83 |
| Gamma-Glutamyl-2-aminobutyric acid | C9 H16 N2 O5 | -0.56 | 232.1058 | 233.1131 | [M+H]+1 | 23.45 |
| Gamma-Glutamyltyrosine | C14 H18 N2 O6 | 0.75 | 310.1167 | 291.0989 | [M-H-H2O]-1 | 2.49 |
| Gln-Gln | C10 H18 N4 O5 | -0.51 | 274.1276 | 275.1349 | [M+H]+1 | 24.00 |
| Gln-Glu | C10 H17 N3 O6 | 0.51 | 275.1119 | 276.119 | [M+H]+1 | 24.29 |
| Glu-Asn | C9 H15 N3 O6 | 0.05 | 261.0961 | 260.089 | [M-H]-1 | 24.78 |
| glu-asp | C9 H14 N2 O7 | 0.42 | 262.0802 | 261.073 | [M-H]-1 | 23.61 |
| Glu-Gln | C10 H17 N3 O6 | -0.07 | 275.1117 | 258.1084 | [M+H-H2O]+1 | 23.53 |
| Glu-Gly | C7 H12 N2 O5 | -1.22 | 204.0744 | 205.0817 | [M+H]+1 | 20.85 |
| Glutamyl pyruvate | C8 H11 N O6 | -0.19 | 217.0586 | 218.0659 | [M+H]+1 | 24.99 |
| Î³-Glutamylaspartic acid | C9 H14 N2 O7 | -1.19 | 262.0798 | 263.0871 | [M+H]+1 | 23.06 |
| Ile-Glu | C11 H20 N2 O5 | 0.23 | 260.1373 | 259.13 | [M-H]-1 | 20.55 |
| L-Aspartic acid | C4 H7 N O4 | 0.66 | 133.0376 | 132.0303 | [M-H]-1 | 22.56 |
| Leu-Arg | C12 H25 N5 O3 | -0.9 | 287.1955 | 288.2028 | [M+H]+1 | 25.49 |
| Leucyltryptophan | C17 H23 N3 O3 | -1.07 | 317.1736 | 318.1809 | [M+H]+1 | 2.94 |
| Leu-Phe | C15 H22 N2 O3 | -0.22 | 278.163 | 279.1703 | [M+H]+1 | 2.85 |
| Leu-Thr | C10 H20 N2 O4 | -0.22 | 232.1423 | 231.135 | [M-H]-1 | 9.79 |
| Leu-Val | C11 H22 N2 O3 | -0.42 | 230.163 | 229.1557 | [M-H]-1 | 5.22 |
| L-Tryptophan | C11 H12 N2 O2 | 0.87 | 204.0901 | 203.0828 | [M-H]-1 | 4.84 |
| N2-Acetylornithine | C7 H14 N2 O3 | 0.2 | 174.1005 | 173.0932 | [M-H]-1 | 21.24 |
| N-Acetylglutamic acid | C7 H11 N O5 | -0.29 | 189.0637 | 172.0604 | [M+H-H2O]+1 | 7.31 |
| N-Acetyl-L-asparagine | C6 H10 N2 O4 | 0.17 | 174.0641 | 173.0568 | [M-H]-1 | 8.16 |
| N-Acetyl-L-aspartic acid | C6 H9 N O5 | 0.56 | 175.0482 | 174.0409 | [M-H]-1 | 9.02 |
| N-Acetyl-L-histidine | C8 H11 N3 O3 | -0.15 | 197.08 | 196.0728 | [M-H]-1 | 15.61 |
| N-Acetyl-L-phenylalanine | C11 H13 N O3 | 0.04 | 207.0896 | 206.0823 | [M-H]-1 | 6.05 |
| N-Acetyltryptophan | C13 H14 N2 O3 | 0.02 | 246.1005 | 245.0932 | [M-H]-1 | 5.98 |
| O-Acetylserine | C5 H9 N O4 | 0.53 | 147.0532 | 146.046 | [M-H]-1 | 5.02 |
| Ophthalmic acid | C11 H19 N3 O6 | 0.98 | 289.1277 | 288.1204 | [M-H]-1 | 22.54 |
| Pro-gln | C10 H17 N3 O4 | -1.01 | 243.1217 | 244.1289 | [M+H]+1 | 23.51 |
| Val-Asp | C9 H16 N2 O5 | 0.21 | 232.106 | 231.0987 | [M-H]-1 | 22.54 |
| Val-Glu | C10 H18 N2 O5 | 0.16 | 246.1216 | 245.1143 | [M-H]-1 | 22.51 |
| Val-Ile | C11 H22 N2 O3 | -1.33 | 230.1627 | 231.17 | [M+H]+1 | 5.15 |
| Val-Met | C10 H20 N2 O3 S | -0.81 | 248.1193 | 249.1264 | [M+H]+1 | 6.14 |
| D-Raffinose pentahydrate | C18 H32 O16 | -0.59 | 504.1687 | 543.1319 | [M+K]+1 | 20.64 |
| D-Ribose | C5 H10 O5 | 0.45 | 150.0529 | 131.0351 | [M-H-H2O]-1 | 3.98 |
| Galactitol | C6 H14 O6 | 0.59 | 182.0792 | 181.0719 | [M-H]-1 | 5.47 |
| Maltotriose | C18 H32 O16 | -0.15 | 504.169 | 503.1617 | [M-H]-1 | 24.26 |
| Sucrose | C12 H22 O11 | 0.19 | 342.1163 | 341.1091 | [M-H]-1 | 14.41 |
| 12-Oxo phytodienoic acid | C18 H28 O3 | -0.91 | 292.2036 | 275.2004 | [M+H-H2O]+1 | 0.83 |
| alpha-Linolenic acid | C18 H30 O2 | -2.13 | 278.224 | 320.2578 | [M+ACN+H]+1 | 0.84 |
| (-)-Epigallocatechin | C15 H14 O7 | -0.67 | 306.0738 | 635.1367 | [2M+Na]+1 | 4.43 |
| 3'.4'.7-Trihydroxyflavanone | C15 H12 O5 | -0.92 | 272.0682 | 273.0755 | [M+H]+1 | 4.40 |
| Isorhamnetin | C16 H12 O7 | 0.38 | 316.0584 | 315.0512 | [M-H]-1 | 0.82 |
| 3'.4'.5.7-Tetrahydroxyisoflavanone | C15 H12 O6 | -0.9 | 288.0631 | 289.0704 | [M+H]+1 | 3.57 |
| 7-Hydroxy-2-(4-hydroxyphenyl)-4-oxo-3.4-dihydro-2H-chromen-5-yl ß-D-glucopyranoside | C21 H22 O10 | -0.16 | 434.1212 | 433.114 | [M-H]-1 | 1.25 |
| ACETYLGLYCITIN | C24 H24 O11 | 0.1 | 488.1319 | 487.1246 | [M-H]-1 | 5.06 |
| Calycosin | C16 H12 O5 | -0.43 | 284.0684 | 283.0613 | [M-H]-1 | 1.15 |
| Catechin | C15 H14 O6 | 0.79 | 290.0793 | 289.072 | [M-H]-1 | 0.91 |
| Coumestrol | C15 H8 O5 | -1.09 | 268.0369 | 269.0442 | [M+H]+1 | 7.07 |
| Daidzein | C15 H10 O4 | -1.14 | 254.0576 | 255.0649 | [M+H]+1 | 0.82 |
| Daidzein-4'-glucoside | C21 H20 O9 | -1.39 | 416.1102 | 417.1174 | [M+H]+1 | 1.41 |
| Daidzin | C21 H20 O9 | -1.23 | 416.1102 | 417.1175 | [M+H]+1 | 1.04 |
| Daidzoside | C21 H20 O9 | 0 | 416.1107 | 415.1035 | [M-H]-1 | 9.80 |
| Genistein | C15 H10 O5 | 0.51 | 270.053 | 269.0457 | [M-H]-1 | 2.33 |
| Malonyldaidzin | C24 H22 O12 | -0.96 | 502.1106 | 503.1179 | [M+H]+1 | 1.75 |
| Malonylgenistin | C24 H22 O13 | -1.13 | 518.1055 | 519.1127 | [M+H]+1 | 2.32 |
| Glycerol monopalmitate | C19 H38 O4 | -1.14 | 330.2766 | 313.2734 | [M+H-H2O]+1 | 0.77 |
| 2'-Deoxyadenosine | C10 H13 N5 O3 | -1.22 | 251.1015 | 252.1088 | [M+H]+1 | 1.42 |
| 2'-Deoxyinosine | C10 H12 N4 O4 | -5.01 | 252.0846 | 251.0773 | [M-H]-1 | 9.00 |
| 5.6-Dihydrouridine | C9 H14 N2 O6 | 0.07 | 246.0852 | 245.0779 | [M-H]-1 | 9.98 |
| Adenosine | C10 H13 N5 O4 | -0.9 | 267.0965 | 268.1038 | [M+H]+1 | 1.68 |
| Deoxyinosine | C10 H12 N4 O4 | -4.95 | 252.0846 | 251.0773 | [M-H]-1 | 6.10 |
| Guanosine | C10 H13 N5 O5 | -0.2 | 283.0916 | 322.0546 | [M+K]+1 | 3.83 |
| Uridine | C9 H12 N2 O6 | -0.2 | 244.0695 | 243.0622 | [M-H]-1 | 3.20 |
| cIMP | C10 H11 N4 O7 P | -3.86 | 330.0353 | 329.028 | [M-H]-1 | 22.00 |
| Nicotinamide adenine dinucleotide (NAD+) | C21 H27 N7 O14 P2 | -0.84 | 663.1086 | 664.1157 | [M+H]+1 | 24.07 |
| Uridine 5'-diphosphate | C9 H14 N2 O12 P2 | -1.13 | 404.0017 | 405.009 | [M+H]+1 | 23.86 |
| Ascorbic acid | C6 H8 O6 | 0.34 | 176.0322 | 175.0249 | [M-H]-1 | 23.72 |
| Citric Acid | C6 H8 O7 | 0.89 | 192.0272 | 191.0199 | [M-H]-1 | 22.59 |
| Dehydroascorbic acid | C6 H6 O6 | -0.21 | 174.0164 | 173.0091 | [M-H]-1 | 1.49 |
| D-Glyceric acid | C3 H6 O4 | 0.66 | 106.0267 | 105.0194 | [M-H]-1 | 5.45 |
| Pyruvic acid | C3 H4 O3 | 0.77 | 88.01611 | 87.00883 | [M-H]-1 | 3.99 |
| Trans-aconitic acid | C6 H6 O6 | -0.3 | 174.0164 | 173.0091 | [M-H]-1 | 2.24 |
| Adenine | C5 H5 N5 | -0.34 | 135.0545 | 136.0617 | [M+H]+1 | 1.55 |
| Guanine | C5 H5 N5 O | -0.5 | 151.0493 | 152.0566 | [M+H]+1 | 8.49 |
| Cytosine | C4 H5 N3 O | -0.01 | 111.0433 | 112.0505 | [M+H]+1 | 2.34 |
| 1-O-(4-Hydroxybenzoyl)-ß-D-glucopyranose | C13 H16 O8 | 0.2 | 300.0846 | 299.0773 | [M-H]-1 | 4.15 |
| 2-Hydroxycinnamic acid | C9 H8 O3 | 0.11 | 164.0474 | 163.0401 | [M-H]-1 | 1.86 |
| 3-Hydroxycinnamic acid | C9 H8 O3 | -0.49 | 164.0473 | 147.044 | [M+H-H2O]+1 | 1.47 |
| Homogentisic acid | C8 H8 O4 | 0.22 | 168.0423 | 167.035 | [M-H]-1 | 3.32 |
| Mevalonic acid | C6 H12 O4 | 0.12 | 148.0736 | 147.0663 | [M-H]-1 | 2.36 |
| 4-Pyridoxic acid | C8 H9 N O4 | -0.58 | 183.0531 | 201.0868 | [M+NH4]+1 | 6.41 |

**Table 3S**

| **Chemical Species** | **Formulae** | **Annot.**  **∆Mass [ppm]** | **Calc.**  **MW** | **m/z** | **Reference**  **Ion** | **RT [min]** |
| --- | --- | --- | --- | --- | --- | --- |
| Indole | C8 H7 N | -0.4 | 117.0578 | 118.0651 | [M+H]+1 | 1.07 |
| Trigonelline | C7 H7 N O2 | 0.05 | 137.0477 | 138.055 | [M+H]+1 | 9.83 |
| y-Aminobutyric acid (GABA) | C4 H9 N O2 | 0.28 | 103.0634 | 86.06007 | [M+H-H2O]+1 | 19.76 |
| y-Glu-Ala | C8 H14 N2 O5 | -0.78 | 218.0901 | 217.0828 | [M-H]-1 | 5.05 |
| Aminoadipic acid | C6 H11 N O4 | 0.1 | 161.0688 | 144.0655 | [M+H-H2O]+1 | 3.68 |
| Asn-Ile-Leu | C16 H30 N4 O5 | -10.85 | 358.2177 | 339.1999 | [M-H-H2O]-1 | 0.65 |
| Asn-Thr-Tyr | C17 H24 N4 O7 | -3.97 | 396.1629 | 435.1261 | [M+K]+1 | 2.63 |
| Asp-Glu | C9 H14 N2 O7 | -0.49 | 262.08 | 263.0873 | [M+H]+1 | 23.11 |
| Cys-pro | C8 H14 N2 O3 S | -1.06 | 218.0723 | 219.0796 | [M+H]+1 | 1.49 |
| L-Glutamine | C5 H10 N2 O3 | -0.02 | 146.0691 | 147.0764 | [M+H]+1 | 23.08 |
| DL-Pyroglutamic acid | C5 H7 N O3 | 0.56 | 129.0427 | 130.0499 | [M+H]+1 | 23.08 |
| gamma-Glutamyl-2-aminobutyric acid | C9 H16 N2 O5 | -1.35 | 232.1056 | 233.1129 | [M+H]+1 | 23.52 |
| gamma-Glutamyltyrosine | C14 H18 N2 O6 | 0.45 | 310.1166 | 291.0988 | [M-H-H2O]-1 | 2.51 |
| Gln-Gln | C10 H18 N4 O5 | 0.82 | 274.1279 | 273.1207 | [M-H]-1 | 23.84 |
| Glu-Ala | C8 H14 N2 O5 | -0.83 | 218.0901 | 219.0974 | [M+H]+1 | 23.06 |
| glu-asp | C9 H14 N2 O7 | 0.43 | 262.0802 | 261.0729 | [M-H]-1 | 23.73 |
| Glu-Gln | C10 H17 N3 O6 | 0.22 | 275.1118 | 276.1189 | [M+H]+1 | 24.33 |
| Glu-Gly | C7 H12 N2 O5 | -0.84 | 204.0745 | 205.0817 | [M+H]+1 | 23.07 |
| Glycyl-prolyl-glutamic acid | C12 H19 N3 O6 | -0.67 | 301.1272 | 302.1345 | [M+H]+1 | 7.00 |
| Isoleucylproline | C11 H20 N2 O3 | -1.1 | 228.1471 | 229.1544 | [M+H]+1 | 16.58 |
| L-?-Glutamyl-L-valine | C10 H18 N2 O5 | 0.41 | 246.1217 | 245.1144 | [M-H]-1 | 20.75 |
| L-Aspartic acid | C4 H7 N O4 | 0.66 | 133.0376 | 132.0303 | [M-H]-1 | 22.66 |
| L-Glutamic acid | C5 H9 N O4 | -0.27 | 147.0531 | 148.0604 | [M+H]+1 | 3.89 |
| Methionylleucine | C11 H22 N2 O3 S | -0.33 | 262.135 | 263.1423 | [M+H]+1 | 4.34 |
| N-a-Acetyl-L-arginine | C8 H16 N4 O3 | -0.57 | 216.1221 | 217.1294 | [M+H]+1 | 19.38 |
| N-Acetylglutamic acid | C7 H11 N O5 | -0.13 | 189.0637 | 172.0604 | [M+H-H2O]+1 | 7.36 |
| N-Acetyl-L-asparagine | C6 H10 N2 O4 | 0.51 | 174.0642 | 173.0569 | [M-H]-1 | 8.25 |
| N-Acetyl-L-histidine | C8 H11 N3 O3 | 0.05 | 197.0801 | 196.0728 | [M-H]-1 | 15.71 |
| N-Acetyltryptophan | C13 H14 N2 O3 | -0.6 | 246.1003 | 245.093 | [M-H]-1 | 6.15 |
| O-Acetylserine | C5 H9 N O4 | 0.53 | 147.0532 | 146.046 | [M-H]-1 | 5.03 |
| Ophthalmic acid | C11 H19 N3 O6 | 0.45 | 289.1275 | 288.1202 | [M-H]-1 | 22.64 |
| Val-Ile | C11 H22 N2 O3 | -0.93 | 230.1628 | 231.1701 | [M+H]+1 | 5.19 |
| D-Raffinose pentahydrate | C18 H32 O16 | -0.03 | 504.169 | 503.162 | [M-H]-1 | 23.58 |
| D-Glucose | C6 H12 O6 | 0.14 | 180.0634 | 215.0328 | [M+Cl]-1 | 6.12 |
| D-Ribose | C5 H10 O5 | 0.65 | 150.0529 | 131.0351 | [M-H-H2O]-1 | 4.04 |
| Galactitol | C6 H14 O6 | 0.84 | 182.0792 | 181.0719 | [M-H]-1 | 5.46 |
| Sucrose | C12 H22 O11 | 0.09 | 342.1162 | 341.109 | [M-H]-1 | 14.43 |
| 12-Oxo phytodienoic acid | C18 H28 O3 | -1.12 | 292.2035 | 275.2002 | [M+H-H2O]+1 | 0.92 |
| Linoleic acid | C18 H32 O2 | -0.05 | 280.2402 | 279.2329 | [M-H]-1 | 0.78 |
| Traumatic acid | C12 H20 O4 | -0.95 | 228.1359 | 229.1432 | [M+H]+1 | 1.03 |
| 3'.4'.7-Trihydroxyflavanone | C15 H12 O5 | -1.25 | 272.0681 | 273.0754 | [M+H]+1 | 4.39 |
| 3'.4'.5.7-Tetrahydroxyisoflavanone | C15 H12 O6 | -1.01 | 288.0631 | 289.0704 | [M+H]+1 | 3.56 |
| 7-Hydroxy-2-(4-hydroxyphenyl)-4-oxo-3.4-dihydro-2H-chromen-5-yl ß-D-glucopyranoside | C21 H22 O10 | 0.2 | 434.1214 | 433.1141 | [M-H]-1 | 1.05 |
| Calycosin | C16 H12 O5 | 0.61 | 284.0687 | 283.0614 | [M-H]-1 | 1.43 |
| Coumestrol | C15 H8 O5 | 0.5 | 268.0373 | 267.03 | [M-H]-1 | 0.80 |
| Daidzein | C15 H10 O4 | -0.15 | 254.0579 | 253.0507 | [M-H]-1 | 21.55 |
| Daidzoside | C21 H20 O9 | 0.22 | 416.1108 | 415.1036 | [M-H]-1 | 9.86 |
| Genistein | C15 H10 O5 | 0.4 | 270.0529 | 269.0457 | [M-H]-1 | 1.49 |
| Malonyldaidzin | C24 H22 O12 | -0.72 | 502.1108 | 503.118 | [M+H]+1 | 1.49 |
| Malonylgenistin | C24 H22 O13 | -0.89 | 518.1056 | 519.1129 | [M+H]+1 | 1.07 |
| 1.3-dilinolenoylglycerol | C39 H64 O5 | -0.37 | 612.4752 | 613.4824 | [M+H]+1 | 0.99 |
| 3-O-ß-D-galactosyl-sn-glycerol | C9 H18 O8 | -0.08 | 254.1002 | 277.0893 | [M+Na]+1 | 6.55 |
| Glycerophosphoethanolamine | C5 H14 N O6 P | 1.36 | 215.0562 | 214.0489 | [M-H]-1 | 6.46 |
| Glycerophosphoglycerol | C6 H15 O8 P | -0.29 | 246.0504 | 245.0431 | [M-H]-1 | 12.10 |
| PC(20:0/P-18:0) | C52 H80 N O8 P | 4.9 | 877.5665 | 878.5737 | [M+H]+1 | 14.06 |
| 2'-Deoxyadenosine | C10 H13 N5 O3 | -0.91 | 251.1016 | 252.1089 | [M+H]+1 | 1.42 |
| 2'-Deoxyinosine | C10 H12 N4 O4 | -5.07 | 252.0846 | 251.0773 | [M-H]-1 | 9.03 |
| Adenosine | C10 H13 N5 O4 | -0.67 | 267.0966 | 268.1039 | [M+H]+1 | 1.66 |
| Guanosine | C10 H13 N5 O5 | -0.92 | 283.0914 | 322.0544 | [M+K]+1 | 3.85 |
| Pseudouridine | C9 H12 N2 O6 | 0.11 | 244.0696 | 243.0623 | [M-H]-1 | 3.21 |
| Uridine | C9 H12 N2 O6 | 0.96 | 244.0698 | 243.0626 | [M-H]-1 | 1.64 |
| Cyclic ADP-ribose | C15 H21 N5 O13 P2 | -0.11 | 541.0611 | 540.0538 | [M-H]-1 | 24.08 |
| Nicotinamide adenine dinucleotide (NAD+) | C21 H27 N7 O14 P2 | -0.74 | 663.1086 | 664.1159 | [M+H]+1 | 24.06 |
| UDP-N-acetylglucosamine | C17 H27 N3 O17 P2 | 0.68 | 607.082 | 606.0747 | [M-H]-1 | 22.76 |
| 2-Isopropylmalic acid | C7 H12 O5 | 0.56 | 176.0686 | 175.0613 | [M-H]-1 | 6.19 |
| 2-Methylcitric acid | C7 H10 O7 | 0.71 | 206.0428 | 205.0355 | [M-H]-1 | 23.75 |
| Cis-Aconitic acid | C6 H6 O6 | 0.4 | 174.0165 | 173.0092 | [M-H]-1 | 2.32 |
| Citric acid | C6 H8 O7 | 0.64 | 192.0271 | 226.9965 | [M+Cl]-1 | 1.53 |
| Dehydroascorbic acid | C6 H6 O6 | -0.12 | 174.0164 | 173.0091 | [M-H]-1 | 1.56 |
| D-Glyceric acid | C3 H6 O4 | 0.94 | 106.0267 | 105.0194 | [M-H]-1 | 5.47 |
| Ethylmalonic acid | C5 H8 O4 | 0.74 | 132.0424 | 131.0351 | [M-H]-1 | 16.65 |
| Glucaric acid | C6 H10 O8 | 0.72 | 210.0377 | 209.0304 | [M-H]-1 | 22.71 |
| Pimelic acid | C7 H12 O4 | -0.04 | 160.0736 | 159.0663 | [M-H]-1 | 1.53 |
| Pyruvic acid | C3 H4 O3 | 0.68 | 88.0161 | 87.00883 | [M-H]-1 | 3.89 |
| Adenine | C5 H5 N5 | 0.19 | 135.0545 | 136.0618 | [M+H]+1 | 1.59 |
| Guanine | C5 H5 N5 O | -0.2 | 151.0494 | 152.0567 | [M+H]+1 | 8.49 |
| 1-Methylcytosine | C5 H7 N3 O | 0.4 | 125.059 | 126.0662 | [M+H]+1 | 6.03 |
| Cytosine | C4 H5 N3 O | 0.54 | 111.0433 | 112.0506 | [M+H]+1 | 3.13 |
| Dihydrothymine | C5 H8 N2 O2 | 0.02 | 128.0586 | 129.0659 | [M+H]+1 | 2.50 |
| 2-hydroxy-5-[3.4.5-trihydroxy-6-(hydroxymethyl)oxan-2-yl]oxybenzoic acid | C13 H16 O9 | 0.35 | 316.0795 | 315.0723 | [M-H]-1 | 2.49 |
| 2-Hydroxycinnamic acid | C9 H8 O3 | 0.48 | 164.0474 | 163.0402 | [M-H]-1 | 2.78 |
| 3-Methoxy-4-[3.4.5-trihydroxy-6-(hydroxymethyl)oxan-2-yl]oxybenzoic acid | C14 H18 O9 | -0.69 | 330.0949 | 353.0841 | [M+Na]+1 | 9.80 |
| 4-(beta-D-Glucopyranosyloxy)phenylacetic acid | C14 H18 O8 | -0.79 | 314.0999 | 337.0891 | [M+Na]+1 | 6.13 |
| 4-Aminobenzoic acid (PABA) | C7 H7 N O2 | -0.17 | 137.0477 | 138.0549 | [M+H]+1 | 1.15 |
| 4-Hydroxycinnamic acid | C9 H8 O3 | 0.66 | 164.0475 | 163.0402 | [M-H]-1 | 1.89 |
| 4'-O-beta-D-glucosyl-cis-p-coumaric acid | C15 H18 O8 | 0.32 | 326.1003 | 361.0698 | [M+Cl]-1 | 2.80 |
| 5-Hydroxybenzofuran-2(3H)-one | C8 H6 O3 | -0.4 | 150.0316 | 151.0389 | [M+H]+1 | 3.36 |
| Phloroglucinol | C6 H6 O3 | 0.3 | 126.0317 | 127.039 | [M+H]+1 | 2.63 |
| Oleanolic acid | C30 H48 O3 | 0.38 | 456.3605 | 455.3532 | [M-H]-1 | 0.79 |
| Pyridoxal | C8 H9 N O3 | -0.36 | 167.0582 | 150.0549 | [M+H-H2O]+1 | 1.32 |

**Table 4S**

| **Chemical species** | **Annot. ∆Mass [ppm]** | **Calc. MW** | **m/z** | **Reference Ion** | **RT [min]** | **MS2 Purity [%]** |
| --- | --- | --- | --- | --- | --- | --- |
| C7 H14 Mn N3 O4 P | -1.34 | 290.0099 | 143.9977 | [M-2H]-2 | 0.66 | 100 |
| C22 H41 O3 P S | -1.37 | 416.2508 | 831.4944 | [2M-H]-1 | 0.72 | 100 |
| C15 H29 N3 O7 S2 | -2.99 | 427.1434 | 426.1361 | [M-H]-1 | 0.74 | 100 |
| C16 H31 N3 O7 S2 | -3.29 | 441.1589 | 440.1516 | [M-H]-1 | 0.78 | 100 |
| C45 H68 N2 O12 | -0.01 | 828.4772 | 829.4845 | [M+H]+1 | 0.82 | 100 |
| C40 H82 Mn N2 O13 | -0.01 | 853.5197 | 852.5125 | [M-H]-1 | 0.83 | 100 |
| C12 H31 N4 O4 P | 2.26 | 326.209 | 325.2018 | [M-H]-1 | 0.87 | 99.8 |
| C34 H72 Mn N2 O15 | -0.04 | 803.4313 | 802.424 | [M-H]-1 | 0.92 | 100 |
| C34 H72 Mn N O7 P | -0.42 | 692.4424 | 691.4351 | [M-H]-1 | 0.97 | 100 |
| C32 H64 N8 O4 P2 | 0.38 | 686.4528 | 685.4456 | [M-H]-1 | 0.98 | 100 |
| C32 H67 N6 O9 P S | -0.09 | 742.4427 | 723.4251 | [M-H-H2O]-1 | 0.99 | 100 |
| C30 H67 N6 O8 P S | -0.1 | 702.4478 | 701.4405 | [M-H]-1 | 0.99 | 100 |
| C32 H64 N8 O6 P2 | 0.34 | 718.4427 | 717.4353 | [M-H]-1 | 0.99 | 97.7 |
| C34 H72 Mn N O10 P | -0.21 | 740.4273 | 739.4201 | [M-H]-1 | 0.99 | 97.8 |
| C23 H41 N4 O5 P | -2.42 | 484.2803 | 483.273 | [M-H]-1 | 0.99 | 97.2 |
| C22 H20 N4 O6 | -2.72 | 436.1371 | 435.1298 | [M-H]-1 | 1.03 | 100 |
| C21 H23 N O13 | 0.29 | 497.1171 | 478.0993 | [M-H-H2O]-1 | 1.04 | 100 |
| C18 H21 N8 O5 P | -0.14 | 460.1372 | 459.1299 | [M-H]-1 | 1.04 | 93.5 |
| C14 H31 N O2 | -0.39 | 245.2354 | 246.2427 | [M+H]+1 | 1.05 | 100 |
| C18 H26 N4 O6 | -2.73 | 394.1842 | 393.1769 | [M-H]-1 | 1.05 | 100 |
| C29 H21 N5 O5 P2 | 0.15 | 581.1019 | 580.0946 | [M-H]-1 | 1.06 | 98.1 |
| C52 H84 N2 O9 P2 S | 0 | 974.5373 | 975.5446 | [M+H]+1 | 1.07 | 100 |
| C27 H17 N4 O4 P3 | -0.13 | 554.0462 | 555.0535 | [M+H]+1 | 1.08 | 100 |
| C56 H51 N2 O5 P3 S3 | 0 | 1020.217 | 1019.21 | [M-H]-1 | 1.14 | 100 |
| C24 H23 N O15 | 0.1 | 565.1068 | 564.0996 | [M-H]-1 | 1.15 | 100 |
| C11 H25 N8 O5 P | -1.02 | 380.1682 | 379.1609 | [M-H]-1 | 1.19 | 100 |
| C17 H42 Mn N5 O10 P | 0.68 | 562.2054 | 543.1874 | [M-H-H2O]-1 | 1.20 | 100 |
| C21 H25 N8 O11 P S | 0.21 | 628.1102 | 627.103 | [M-H]-1 | 1.46 | 100 |
| C14 H21 N O6 | -1.12 | 299.1366 | 300.1438 | [M+H]+1 | 1.52 | 100 |
| C34 H25 N8 O3 P S | 0.11 | 656.1509 | 657.1581 | [M+H]+1 | 1.52 | 100 |
| C24 H26 O6 S2 | -1.35 | 474.1164 | 473.1092 | [M-H]-1 | 1.54 | 100 |
| C27 H27 O3 P3 S2 | 0.23 | 556.0616 | 557.0689 | [M+H]+1 | 1.55 | 100 |
| C16 H28 O10 | 0.27 | 380.1684 | 379.1611 | [M-H]-1 | 1.58 | 100 |
| C21 H28 Mn N O7 P3 | 0.17 | 554.046 | 555.0533 | [M+H]+1 | 1.61 | 100 |
| C23 H23 Mn N5 O S | 0.2 | 472.1005 | 471.0932 | [M-H]-1 | 1.62 | 100 |
| C25 H25 Mn N5 O4 S | -0.22 | 546.1007 | 547.1079 | [M+H]+1 | 1.64 | 100 |
| C15 H35 Mn N7 P2 S | -0.28 | 462.1529 | 463.1602 | [M+H]+1 | 1.77 | 100 |
| C30 H37 N O15 P2 | -0.01 | 713.1638 | 712.1566 | [M-H]-1 | 1.86 | 100 |
| C6 H12 N8 O4 | 1.79 | 260.0986 | 259.0913 | [M-H]-1 | 18.72 | 93.4 |
| C7 H18 N3 O8 P | 0.68 | 303.0834 | 302.0761 | [M-H]-1 | 18.85 | 100 |
| C22 H24 N4 O9 S | 0.98 | 520.1269 | 559.0903 | [M+K]+1 | 18.91 | 100 |
| C9 H23 N4 O3 P S2 | -1.93 | 330.0943 | 329.087 | [M-H]-1 | 18.98 | 100 |
| C16 H39 Mn N3 O6 P2 | 0.6 | 486.1698 | 485.1625 | [M-H]-1 | 19.33 | 100 |
| C19 H45 N7 O7 S2 | 2.83 | 547.2837 | 548.291 | [M+H]+1 | 19.35 | 100 |
| C24 H49 N O15 | -0.4 | 591.31 | 592.3173 | [M+H]+1 | 19.39 | 100 |
| C15 H37 Mn N3 O6 P2 | 0.09 | 472.1539 | 471.1466 | [M-H]-1 | 19.54 | 100 |
| C18 H30 N6 O10 | -2.3 | 490.2012 | 489.1939 | [M-H]-1 | 20.08 | 94.8 |
| C16 H32 N2 O14 | 0.43 | 476.1856 | 475.1783 | [M-H]-1 | 20.09 | 100 |
| C8 H14 O9 | 0.47 | 254.0639 | 253.0566 | [M-H]-1 | 20.18 | 100 |
| C14 H34 N8 O5 P2 S | 1.22 | 488.1854 | 487.1781 | [M-H]-1 | 20.36 | 100 |
| C25 H41 N4 O11 P S | -0.16 | 636.2229 | 635.2156 | [M-H]-1 | 20.77 | 100 |
| C17 H30 N5 O7 P | -4.24 | 447.1864 | 448.1937 | [M+H]+1 | 20.83 | 100 |
| C12 H23 N O14 | 0.17 | 405.1119 | 404.1046 | [M-H]-1 | 21.11 | 100 |
| C25 H43 N2 O5 P3 S2 | 0.37 | 608.1829 | 609.1901 | [M+H]+1 | 21.31 | 100 |
| C18 H26 N3 O4 P S2 | -0.98 | 443.1098 | 444.1169 | [M+H]+1 | 21.31 | 100 |
| C14 H24 Mn N6 O7 | 2.59 | 443.1099 | 442.1026 | [M-H]-1 | 21.35 | 100 |
| C15 H29 O17 P | 0.37 | 512.1144 | 513.1217 | [M+H]+1 | 21.77 | 100 |
| C15 H28 O13 | 0.44 | 416.1532 | 451.1227 | [M+Cl]-1 | 22.19 | 100 |

**Table 5s**

| **Chemical species** | **Annot. ∆Mass [ppm]** | **Calc. MW** | **m/z** | **Reference Ion** | **RT [min]** | **Polarity** | **MS2 Purity [%]** |
| --- | --- | --- | --- | --- | --- | --- | --- |
| C35 H33 N5 O5 P2 S | 0.05 | 697.1678 | 696.1605 | [M-H]-1 | 1.8 | Negative | 100 |
| C9 H15 N8 O4 P | -3.16 | 330.0943 | 329.0871 | [M-H]-1 | 18.8 | Negative | 100 |
| C10 H25 N2 O10 P S2 | -0.27 | 428.0687 | 427.0614 | [M-H]-1 | 2.1 | Negative | 100 |
| C7 H12 O8 | 0.56 | 224.0533 | 223.0461 | [M-H]-1 | 23.6 | Negative | 100 |
| C18 H21 N4 O12 P | 0.68 | 516.0897 | 517.0970 | [M+H]+1 | 1.6 | Positive | 100 |
| C22 H20 N4 O6 | -3.14 | 436.1369 | 435.1296 | [M-H]-1 | 1.0 | Negative | 100 |
| C7 H12 O8 | 0.63 | 224.0534 | 223.0461 | [M-H]-1 | 18.6 | Negative | 100 |
| C22 H22 Mn N5 O5 P S | -0.16 | 554.0459 | 555.0532 | [M+H]+1 | 1.1 | Positive | 100 |
| C24 H23 N O15 | -0.01 | 565.1068 | 564.0995 | [M-H]-1 | 1.1 | Negative | 100 |
| C25 H18 N2 O9 S2 | 0.05 | 554.0454 | 555.0527 | [M+H]+1 | 1.6 | Positive | 100 |
| C10 H20 N8 O6 S2 | 0.86 | 412.0951 | 411.0878 | [M-H]-1 | 2.1 | Negative | 100 |
| C9 H16 N8 O8 | 1.6 | 364.1097 | 363.1024 | [M-H]-1 | 18.3 | Negative | 100 |
| C18 H18 N6 P2 S | 1.1 | 412.0793 | 471.0932 | [M-H+HAc]-1 | 1.1 | Negative | 100 |
| C7 H12 O8 | 0.27 | 224.0533 | 223.0460 | [M-H]-1 | 22.6 | Negative | 100 |
| C14 H32 N8 O5 P2 S | 1.34 | 486.1698 | 485.1625 | [M-H]-1 | 19.4 | Negative | 100 |
| C6 H18 N5 O6 P | 1.82 | 287.1000 | 288.1073 | [M+H]+1 | 2.7 | Positive | 100 |
| C11 H18 N6 O4 S | -2.17 | 330.1103 | 331.1176 | [M+H]+1 | 1.6 | Positive | 100 |
| C24 H28 O6 S2 | -1.72 | 476.1319 | 475.1245 | [M-H]-1 | 1.2 | Negative | 100 |
| C12 H21 N O11 | -0.05 | 355.1114 | 354.1043 | [M-H]-1 | 28.8 | Both | 100 |
| C26 H25 N7 O9 | -0.72 | 579.1710 | 579.1704 | [M-e]+1 | 4.1 | Positive | 100 |
| C28 H43 Mn N O4 P2 | 0.52 | 574.2051 | 573.1979 | [M-H]-1 | 1.0 | Negative | 100 |
| C21 H22 Mn N6 O3 S | 0.69 | 493.0858 | 492.0785 | [M-H]-1 | 1.0 | Negative | 100 |
| C24 H23 Mn N5 O3 S | -0.5 | 516.0900 | 517.0972 | [M+H]+1 | 2.5 | Positive | 100 |
| C21 H34 N2 O7 P2 | 2.75 | 488.1855 | 487.1782 | [M-H]-1 | 20.3 | Negative | 100 |
| C12 H16 O9 | -0.04 | 304.0794 | 303.0721 | [M-H]-1 | 10.6 | Negative | 100 |
| C14 H34 Mn N2 O4 P2 S | 0.64 | 443.1098 | 442.1025 | [M-H]-1 | 21.3 | Negative | 100 |
| C10 H15 N4 O7 P | -3.51 | 334.0667 | 333.0594 | [M-H]-1 | 22.6 | Negative | 100 |
| C6 H10 N7 O11 P | 1.43 | 387.0181 | 388.0254 | [M+H]+1 | 16.7 | Positive | 100 |
| C10 H15 N4 O7 P | -4.38 | 334.0664 | 333.0594 | [M-H]-1 | 23.0 | Both | 100 |
| C19 H45 Mn N4 O11 P | -0.77 | 591.2198 | 590.2126 | [M-H]-1 | 2.5 | Negative | 100 |
| C26 H37 Mn N O7 P2 | -0.33 | 592.1424 | 593.1497 | [M+H]+1 | 3.3 | Positive | 100 |
| C11 H23 N O13 | 0.13 | 377.1170 | 376.1097 | [M-H]-1 | 9.8 | Negative | 100 |
| C5 H14 N5 O8 P | 0.9 | 303.0583 | 302.0510 | [M-H]-1 | 15.2 | Negative | 100 |
| C18 H29 N3 O10 | 2.45 | 447.1864 | 448.1937 | [M+H]+1 | 20.8 | Positive | 100 |
| C27 H39 Mn N4 O13 P | -0.02 | 713.1632 | 712.1559 | [M-H]-1 | 2.3 | Negative | 100 |
| C2 H7 N4 O6 P | -0.44 | 214.0102 | 215.0175 | [M+H]+1 | 15.1 | Positive | 100 |
| C7 H13 N6 O4 P | -3.42 | 276.0727 | 275.0654 | [M-H]-1 | 16.6 | Negative | 100 |
| C15 H14 N4 O5 | -4 | 330.0951 | 329.0879 | [M-H]-1 | 2.6 | Both | 100 |
| C7 H21 Mn N6 O5 P | 3.83 | 355.0705 | 356.0778 | [M+H]+1 | 17.3 | Positive | 100 |
| C20 H33 N O17 | -0.14 | 559.1748 | 558.1677 | [M-H]-1 | 26.6 | Both | 100 |
| C17 H39 Mn N O9 P2 | 0.77 | 518.1485 | 517.1412 | [M-H]-1 | 26.6 | Negative | 100 |
| C18 H30 O17 | -0.06 | 518.1483 | 517.1410 | [M-H]-1 | 29.1 | Negative | 100 |
| C20 H30 Mn N7 O4 P | 1.14 | 518.1483 | 517.1411 | [M-H]-1 | 28.0 | Negative | 100 |
| C25 H25 Mn N5 O4 S | -0.4 | 546.1006 | 547.1080 | [M+H]+1 | 1.1 | Positive | 90.17 |
| C20 H16 N4 O6 | 1.76 | 408.1077 | 431.0969 | [M+Na]+1 | 1.0 | Positive | 91.08 |
| C16 H15 N8 O5 P | -0.75 | 430.0900 | 429.0827 | [M-H]-1 | 1.0 | Negative | 91.86 |
| C3 H8 Mn O6 | -0.92 | 194.9700 | 195.9772 | [M+H]+1 | 16.6 | Positive | 93.08 |
| C14 H14 N6 O3 S2 | -2.52 | 378.0559 | 379.0632 | [M+H]+1 | 19.6 | Positive | 93.64 |
| C6 H16 Mn N3 P S | -4.4 | 248.0172 | 249.0245 | [M+H]+1 | 7.3 | Positive | 93.66 |
| C11 H22 O10 | 0.1 | 314.1213 | 349.0907 | [M+Cl]-1 | 9.8 | Both | 94.56 |
| C9 H11 Mn N P2 | 3.22 | 249.9755 | 250.9828 | [M+H]+1 | 8.2 | Positive | 94.72 |
| C13 H16 N4 O8 | -3.34 | 356.0956 | 355.0884 | [M-H]-1 | 23.7 | Negative | 95.93 |
| C2 H4 N8 O4 | 2.17 | 204.0360 | 203.0287 | [M-H]-1 | 17.7 | Negative | 96.01 |
| C5 H10 N O P S | -0.12 | 163.0221 | 164.0293 | [M+H]+1 | 17.5 | Positive | 97.03 |
| C5 H10 N8 O3 | 1.18 | 230.0879 | 229.0806 | [M-H]-1 | 16.8 | Negative | 97.54 |
| C23 H23 Mn N5 O S | 0.59 | 472.1007 | 471.0934 | [M-H]-1 | 7.1 | Negative | 97.67 |
| C11 H23 O8 P S2 | -2.96 | 378.0561 | 379.0634 | [M+H]+1 | 6.0 | Positive | 97.72 |
| C28 H23 Mn O6 P | 0.37 | 541.0615 | 540.0542 | [M-H]-1 | 24.0 | Negative | 97.82 |
| C16 H8 N6 O8 | -0.07 | 412.0403 | 413.0476 | [M+H]+1 | 2.5 | Positive | 97.86 |
| C23 H31 N4 O11 P | 0.68 | 570.1731 | 571.1804 | [M+H]+1 | 1.0 | Positive | 98.18 |
| C8 H11 N4 O5 P S | 5.07 | 306.0203 | 344.9834 | [M+K]+1 | 16.8 | Positive | 98.28 |
| C24 H33 N4 O12 P | 0.43 | 600.1835 | 601.1908 | [M+H]+1 | 1.0 | Positive | 98.36 |
| C8 H18 Mn N P | 4.5 | 214.0567 | 213.0494 | [M-H]-1 | 18.2 | Negative | 98.72 |

**Table 6S**

| **Chemical Species** | **Annot. ∆Mass [ppm]** | **Calc. MW** | **m/z** | **Reference Ion** | **RT [min]** | **Polarity** | **MS2 Purity [%]** |
| --- | --- | --- | --- | --- | --- | --- | --- |
| C6 H9 N3 O3 P2 | 1.01 | 233.0122 | 234.0194 | [M+H]+1 | 2.237 | Positive | 100 |
| C20 H28 N6 O P2 S | 1.92 | 462.1529 | 463.1602 | [M+H]+1 | 1.678 | Positive | 100 |
| C13 H16 N8 O5 S2 | 0.4 | 428.0687 | 427.0614 | [M-H]-1 | 2.139 | Negative | 100 |
| C4 H13 N2 P S2 | -4.31 | 184.025 | 185.0323 | [M+H]+1 | 18.894 | Positive | 100 |
| C10 H24 N2 O4 P2 S | 3.28 | 330.0943 | 329.087 | [M-H]-1 | 18.897 | Negative | 100 |
| C25 H23 N O17 | 0.21 | 609.0967 | 564.0996 | [M-CO2-H]-1 | 1.143 | Negative | 100 |
| C22 H20 N4 O6 | -2.79 | 436.1371 | 435.1298 | [M-H]-1 | 1.03 | Negative | 100 |
| C24 H23 N O16 | 0.13 | 581.1018 | 580.0945 | [M-H]-1 | 1.076 | Negative | 100 |
| C17 H24 N4 O7 | -3.61 | 396.1631 | 395.1558 | [M-H]-1 | 3.314 | Negative | 100 |
| C26 H46 N2 O18 P2 S | 0.03 | 768.1942 | 767.1869 | [M-H]-1 | 14.425 | Negative | 100 |
| C11 H22 Mg O14 P2 | -0.25 | 464.0334 | 465.0407 | [M+H]+1 | 14.408 | Positive | 100 |
| C12 H27 N6 P3 S2 | -1.42 | 412.0946 | 413.1018 | [M+H]+1 | 1.881 | Positive | 100 |
| C17 H32 N2 O14 | 0.67 | 488.1857 | 487.1784 | [M-H]-1 | 19.053 | Negative | 100 |
| C9 H24 N4 O10 S2 | 3.66 | 412.0949 | 411.0876 | [M-H]-1 | 2.164 | Negative | 100 |
| C18 H41 Mn N3 O4 P2 | 2.58 | 480.1965 | 481.2038 | [M+H]+1 | 19.623 | Positive | 100 |
| C19 H21 N6 O6 P3 S | 0.16 | 554.0457 | 555.053 | [M+H]+1 | 1.08 | Positive | 100 |
| C17 H35 Mn N5 O3 P2 | -2.68 | 474.1583 | 513.1215 | [M+K]+1 | 20.552 | Positive | 100 |
| C5 H15 N5 O7 S | -4.72 | 289.0679 | 288.0606 | [M-H]-1 | 18.943 | Negative | 100 |
| C14 H34 Mn N2 O4 P2 S | 1.06 | 443.11 | 442.1027 | [M-H]-1 | 21.291 | Negative | 100 |
| C22 H22 Mn N5 O5 P S | -0.16 | 554.0459 | 555.0532 | [M+H]+1 | 1.583 | Positive | 100 |
| C16 H30 N2 O14 | 0.49 | 474.1699 | 473.1627 | [M-H]-1 | 20.269 | Negative | 100 |
| C22 H24 Mn N5 O5 P S | -0.1 | 556.0616 | 557.0689 | [M+H]+1 | 1.073 | Positive | 100 |
| C44 H38 O12 P2 S | 0.02 | 852.1559 | 851.1486 | [M-H]-1 | 14.437 | Negative | 100 |
| C17 H26 N4 O9 P2 S2 | 0.2 | 556.0618 | 557.069 | [M+H]+1 | 1.479 | Positive | 100 |
| C24 H31 Mn N O6 P2 | -0.48 | 546.1005 | 547.1077 | [M+H]+1 | 1.102 | Positive | 100 |
| C14 H21 Mn N O S | 4.39 | 306.0738 | 635.1368 | [2M+Na]+1 | 4.405 | Positive | 100 |
| C9 H10 N6 O3 P2 | -2.19 | 312.0283 | 313.0356 | [M+H]+1 | 17.163 | Positive | 100 |
| C8 H16 O8 | 1.14 | 240.0848 | 275.0543 | [M+Cl]-1 | 10.628 | Negative | 100 |
| C27 H39 N5 O4 P2 S | 0.21 | 591.2199 | 590.2127 | [M-H]-1 | 2.372 | Negative | 100 |
| C32 H32 Mn N7 O4 P | 0.03 | 664.1634 | 665.1707 | [M+H]+1 | 9.903 | Positive | 100 |
| C12 H17 Mn N O5 | -2.67 | 310.0479 | 309.0406 | [M-H]-1 | 1.571 | Negative | 100 |
| C10 H15 N4 O7 P | -3.51 | 334.0667 | 333.0594 | [M-H]-1 | 22.707 | Negative | 100 |
| C16 H23 Mn N O8 | -2.54 | 412.0794 | 471.0932 | [M-H+HAc]-1 | 1.09 | Negative | 100 |
| C12 H21 N O11 | 0.06 | 355.1115 | 354.1043 | [M-H]-1 | 28.765 | Both | 100 |
| C7 H16 Mn N O7 P S | -1 | 343.9762 | 344.9835 | [M+H]+1 | 16.551 | Positive | 100 |
| C7 H17 Mn N7 O5 | -3.14 | 334.0661 | 335.0734 | [M+H]+1 | 23.06 | Positive | 100 |
| C9 H19 N O8 | 1.02 | 269.1113 | 268.1041 | [M-H]-1 | 16.866 | Negative | 100 |
| C8 H25 N7 O4 P2 S | 1.57 | 377.117 | 376.1097 | [M-H]-1 | 9.78 | Negative | 100 |
| C30 H46 Mn N2 O9 P2 S | -0.06 | 727.1779 | 726.1707 | [M-H]-1 | 1.853 | Negative | 100 |
| C21 H34 N2 O7 P2 | 2.94 | 488.1856 | 487.1783 | [M-H]-1 | 20.255 | Negative | 100 |
| C17 H33 Mn N O4 P2 | 0.51 | 432.1268 | 413.1089 | [M-H-H2O]-1 | 2.647 | Both | 100 |
| C17 H32 N2 O14 | 0.74 | 488.1857 | 487.1784 | [M-H]-1 | 20.535 | Negative | 100 |
| C20 H33 N O17 | -0.14 | 559.1748 | 558.1677 | [M-H]-1 | 26.599 | Both | 100 |
| C20 H30 Mn N7 O4 P | 1.14 | 518.1483 | 517.1411 | [M-H]-1 | 26.689 | Negative | 100 |
| C18 H30 O17 | 0.41 | 518.1485 | 517.1412 | [M-H]-1 | 29.125 | Negative | 100 |
| C13 H22 O4 S2 | -3.85 | 306.0948 | 307.1021 | [M+H]+1 | 2.627 | Positive | 83.33 |
| C3 H3 N2 O6 P S | -3.96 | 225.9441 | 226.9513 | [M+H]+1 | 17.457 | Positive | 85.88 |
| C8 H20 N6 O6 P2 S | 3.09 | 390.0652 | 389.058 | [M-H]-1 | 15.208 | Negative | 88.53 |
| C14 H20 N4 O4 | -3.48 | 308.1474 | 307.1401 | [M-H]-1 | 2.619 | Negative | 88.79 |
| C14 H26 Mg O12 P2 | -0.89 | 472.0746 | 473.0819 | [M+H]+1 | 2.85 | Positive | 88.86 |
| C6 H N8 O3 P | 1.39 | 263.9913 | 264.9986 | [M+H]+1 | 7.381 | Positive | 89.18 |
| C10 H20 O8 | 0.63 | 268.116 | 267.1087 | [M-H]-1 | 3.712 | Negative | 90.49 |
| C13 H16 N4 O8 | -3.17 | 356.0957 | 355.0884 | [M-H]-1 | 25.173 | Negative | 91.50 |
| C15 H32 N2 O11 P2 | -1.06 | 478.1476 | 477.1404 | [M-H]-1 | 1.112 | Negative | 92.39 |
| C24 H21 Mn N7 O P2 | -0.02 | 540.0663 | 579.0295 | [M+K]+1 | 1.24 | Positive | 93.05 |
| C10 H5 N5 O5 | 0.45 | 275.0292 | 293.063 | [M+NH4]+1 | 6.624 | Positive | 94.22 |
| C5 H7 Mn N2 O4 P | 0.32 | 244.9525 | 245.9598 | [M+H]+1 | 5.394 | Positive | 95.03 |
| C18 H17 N8 O6 P | -0.44 | 472.1007 | 471.0934 | [M-H]-1 | 15.405 | Negative | 95.77 |
| C10 H15 N O2 P2 S | -2.36 | 275.0292 | 293.0631 | [M+NH4]+1 | 7.494 | Positive | 96.14 |
| C8 H12 N2 O6 Zn | -0.22 | 295.9986 | 294.9915 | [M-H]-1 | 22.103 | Both | 96.30 |
| C16 H18 N2 O5 P2 S | -1.73 | 412.0405 | 413.0477 | [M+H]+1 | 2.527 | Positive | 96.90 |
| C11 H22 O10 | 0.16 | 314.1214 | 349.0907 | [M+Cl]-1 | 9.804 | Both | 97.09 |
| C4 H12 N2 O2 P2 S | 3.81 | 214.0103 | 215.0176 | [M+H]+1 | 15.245 | Positive | 97.82 |
| C16 H31 Mn N O9 | -1.84 | 436.1371 | 435.1299 | [M-H]-1 | 1.464 | Negative | 98.26 |
| C7 H14 N7 O6 P S2 | -0.73 | 387.0182 | 388.0255 | [M+H]+1 | 16.52 | Positive | 98.92 |
| C15 H22 N4 O7 | -3.58 | 370.1475 | 369.1403 | [M-H]-1 | 7.697 | Both | 99.34 |
| C7 H12 O8 | 0.19 | 224.0533 | 223.046 | [M-H]-1 | 22.721 | Negative | 99.67 |

**Figure captions**

**Fig. 1S.** Identification of the known S-containing metabolite S-Methylglutathione (C11H18N3O6) in transgenic soybean calluses (RR variety) using online integrated HILIC-MS-ESI-HRMS-ICP-MS/MS.

**Fig. 2S.** Identification of the putatively annotated Mg-containing metabolite (C11H23MgO14P2) in transgenic soybean calluses using online integrated HILIC-MS-ESI-HRMS-ICP-MS/MS.

**Fig. 3S.** Identification of the putatively annotated Mn-containing metabolite (C7H17Mn N7O5) in transgenic soybean calluses using online integrated HILIC-MS-ESI-HRMS-ICP-MS/MS.

**Fig. 4S.** Identification of the putatively annotated Zn-containing metabolite (C8H11N2O6Zn) in transgenic soybean calluses using online integrated HILIC-MS-ESI-HRMS-ICP-MS/MS.


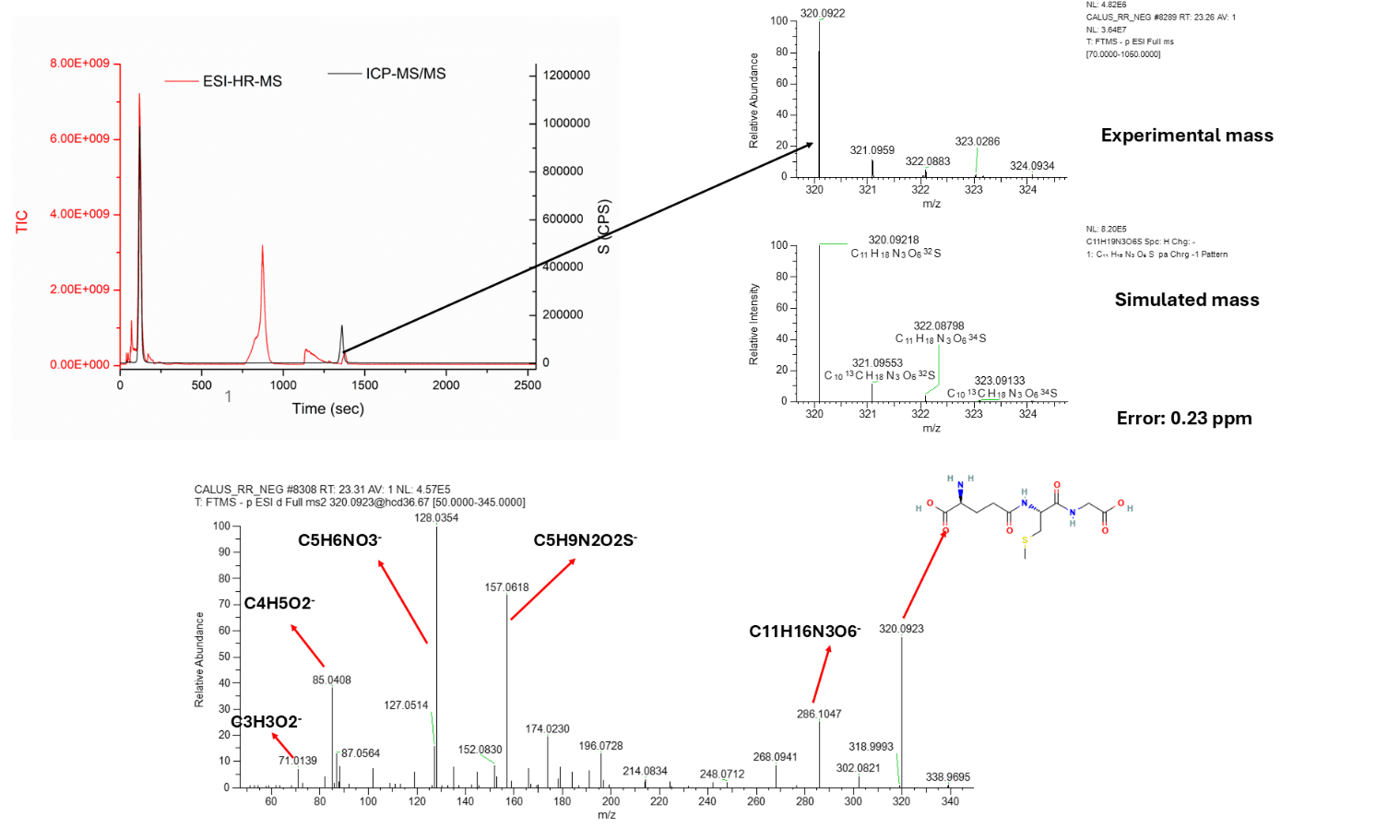


**Fig. 1S**

**
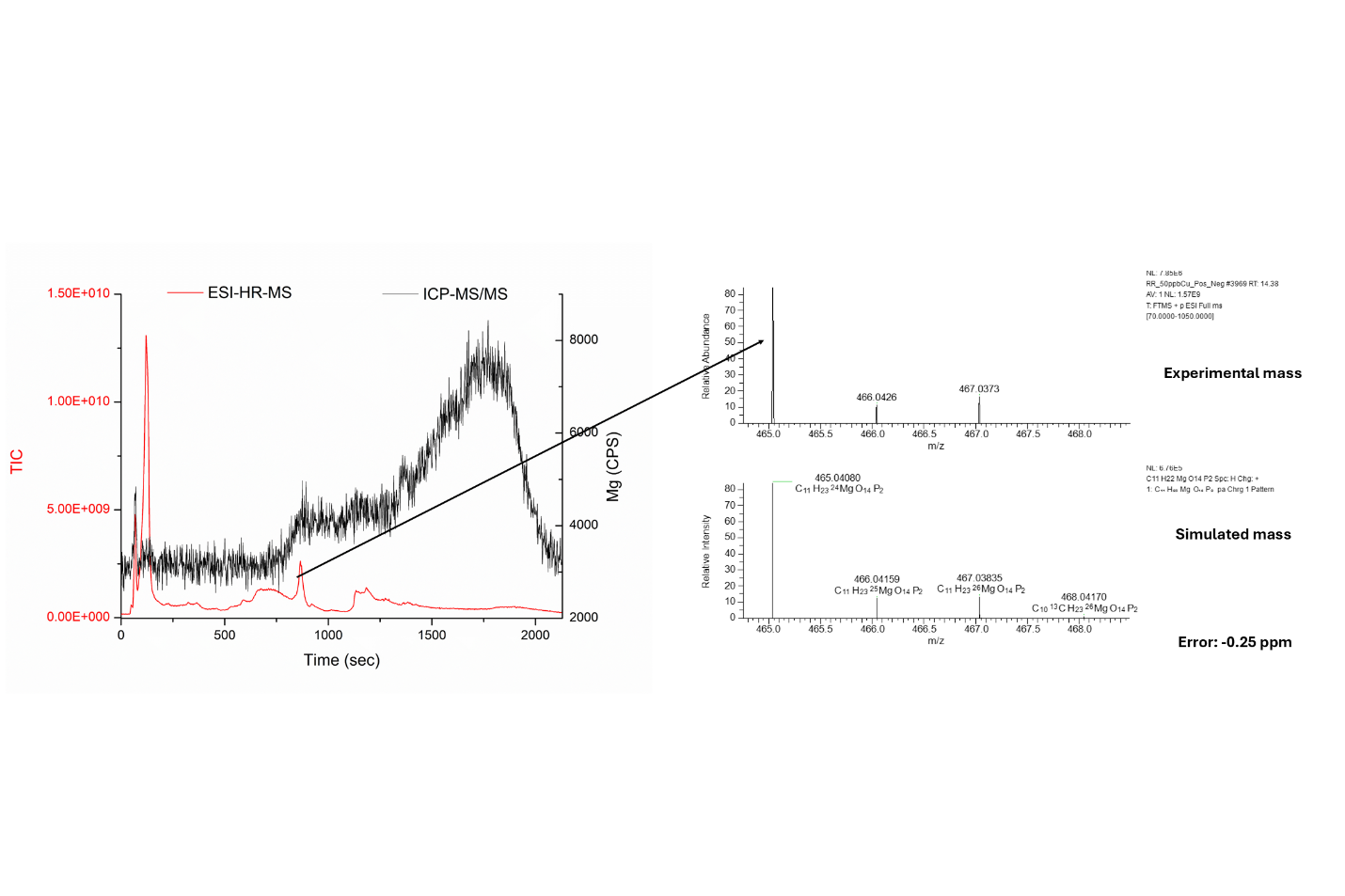
**

**Fig. 2S**

**
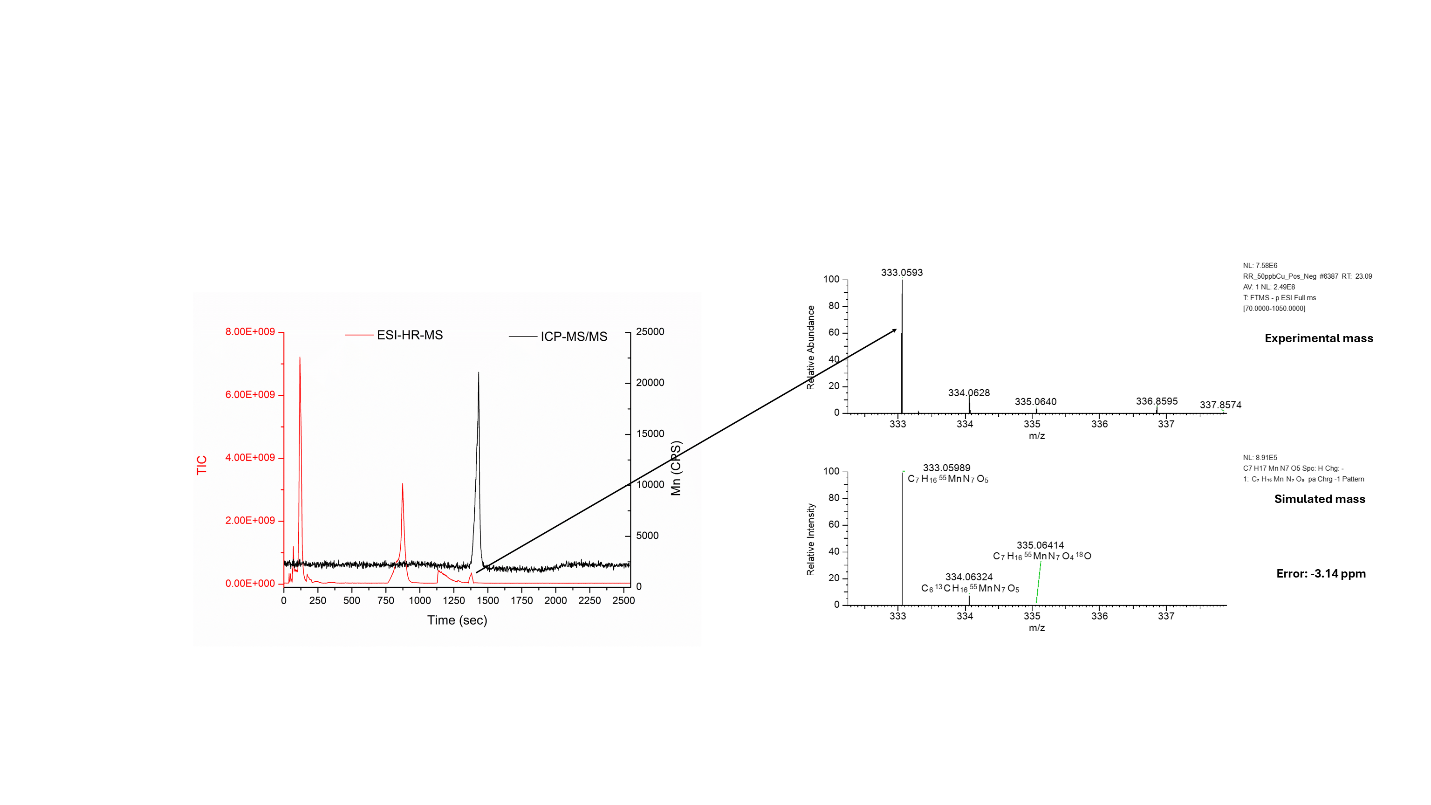
**

**Fig. 3S**

**
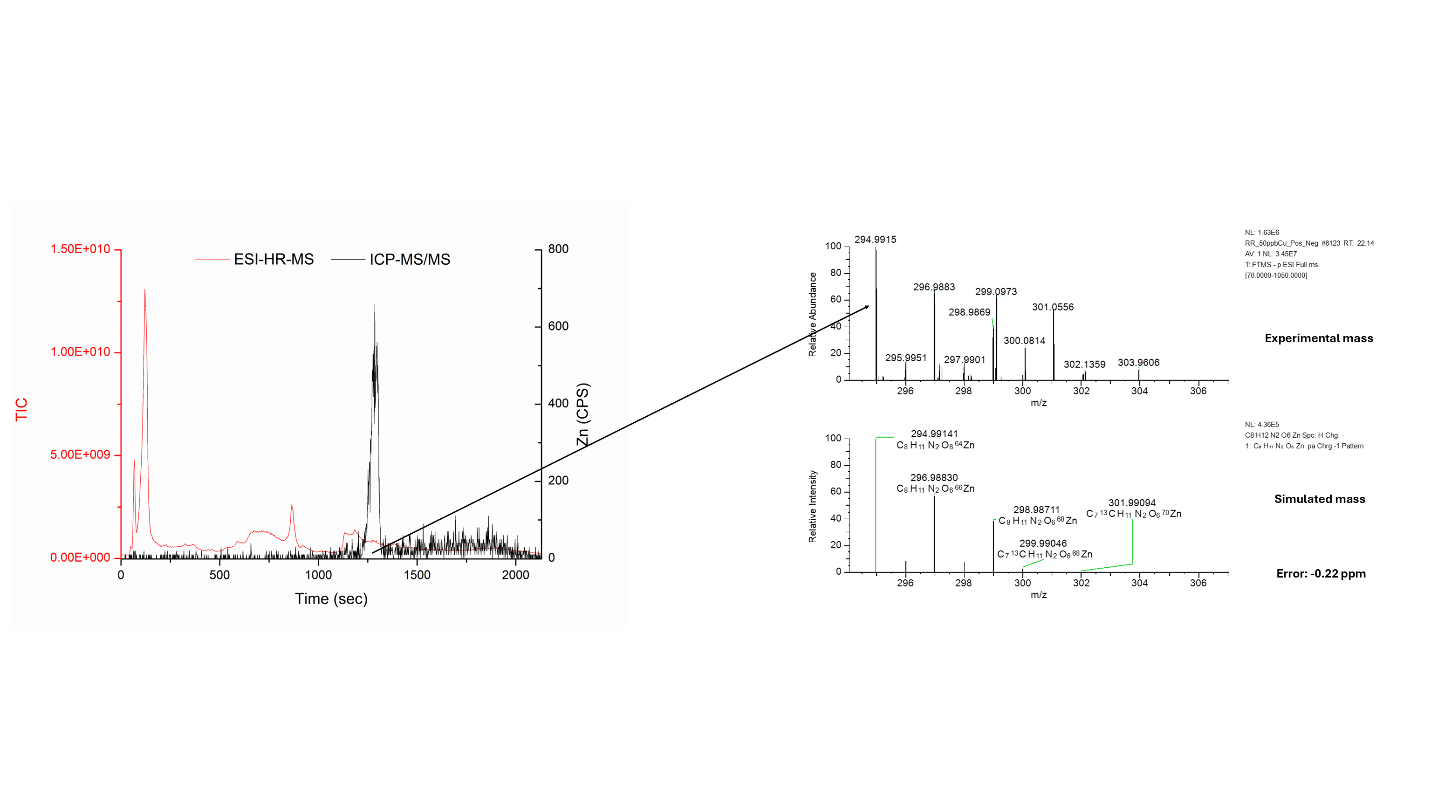
**

**Fig. 4S**
